# *In situ* microscopy of the self-assembly of branched nanocrystals in solution

**DOI:** 10.1038/ncomms11213

**Published:** 2016-04-04

**Authors:** Eli Sutter, Peter Sutter, Alexei V. Tkachenko, Roman Krahne, Joost de Graaf, Milena Arciniegas, Liberato Manna

**Affiliations:** 1 Department of Mechanical and Materials Engineering, University of Nebraska-Lincoln, Lincoln, Nebraska 68588, USA; 2 Department of Electrical and Computer Engineering, University of Nebraska-Lincoln, Lincoln, Nebraska 68588, USA; 3 Center for Functional Nanomaterials, Brookhaven National Laboratory, Upton, New York 11973, USA; 4 Department of Nanochemistry, Istituto Italiano di Tecnologia (IIT), via Morego 30, Genova IT-16163, Italy; 5 Faculty 8: Mathematics and Physics, Institute for Computational Physics (ICP), University of Stuttgart, Allmandring 3, Stuttgart 70569, Germany

## Abstract

Solution-phase self-assembly of nanocrystals into mesoscale structures is a promising strategy for constructing functional materials from nanoscale components. Liquid environments are key to self-assembly since they allow suspended nanocrystals to diffuse and interact freely, but they also complicate experiments. Real-time observations with single-particle resolution could have transformative impact on our understanding of nanocrystal self-assembly. Here we use real-time *in situ* imaging by liquid-cell electron microscopy to elucidate the nucleation and growth mechanism and properties of linear chains of octapod-shaped nanocrystals in their native solution environment. Statistical mechanics modelling based on these observations and using the measured chain-length distribution clarifies the relative importance of dipolar and entropic forces in the assembly process and gives direct access to the interparticle interaction. Our results suggest that monomer-resolved *in situ* imaging combined with modelling can provide unprecedented quantitative insight into the microscopic processes and interactions that govern nanocrystal self-assembly in solution.

## Abstract (web summary)

Understanding the structure and transformation of colloidal matter requires probing configurations from monomers to extended assemblies. Here, the authors use liquid-cell electron microscopy to elucidate the nucleation and growth properties of linear chains of branched nanocrystals in solution.

## Graphical abstract

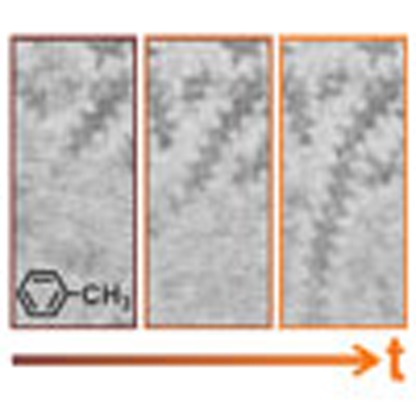

The self-assembly of nanocrystals into ordered superstructures promises access to photonic^1^,^2^, plasmonic^3^, electronic^4^,^5^, magnetic^6^ and catalytic^7^ functionalities that are tunable and may even be reconfigurable in real time^8^. Besides technological interest, research on crystallization of colloids is driven by the recognition that they are not simply large atom equivalents but represent a form of matter with complex collective behaviour that can be controlled via the interplay of entropic, van der Waals, steric and dipolar interactions^9^,^10^,^11^, by attaching molecular linkers such as coordinated ligands or DNA^12^,^13^,^14^, or by using shaped particles^15^. Understanding the structure, formation and transformation of colloidal matter requires probing particle configurations from monomers to extended periodic or aperiodic assemblies. Computer simulations provide this capability^16^,^17^, but experiments are complicated by the need to interrogate processes in liquids that allow suspended particles to diffuse and interact freely. Larger micro-colloids can be imaged with single-particle resolution by optical microscopy^18^,^19^, but offer limited diversity of materials and shapes^20^. For nanocrystals, *in situ* measurements have largely been restricted to reciprocal space techniques^14^,^21^, which yield little information on the assembly process or the underlying forces. Liquid-cell electron microscopy (LCEM) has recently been developed into a tool capable of imaging processes in liquids with nanometre resolution^22^,^23^,^24^. LCEM has been used previously to study single-layer assembly of compact nanocrystals driven by convective transport during solvent drying^25^. A large class of self-assembly processes, for instance the entropy-driven assembly of polyhedral nanocrystals^15^ or the DNA-guided crystallization^12^,^13^,^14^,^26^ and reconfiguration^27^ of spherical nucleic acid conjugates occurs in bulk solutions with transport by Brownian motion.

Using the example of the one-dimensional (1D) self-assembly of CdSe/CdS octapods, we demonstrate the ability of LCEM to dynamically probe such solution-phase self-assembly processes in real space with nanometre resolution. Octapods, branched nanocrystals with a complex but still highly symmetric shape^28^,^29^,^30^, have been found to self-assemble into a variety of superstructures: interlocked 1D chains in bulk liquid, two-dimensional square lattices on substrates^31^ or micron-sized three-dimensional (3D) structures of aligned 1D chains^21^. Strongly anisotropic interactions are thought to be key to their rich set of accessible superstructures. By real-time imaging with monomer resolution, the *in situ* LCEM observations reported here establish several key aspects of the self-assembly of ordered linear chains of octapods in solution. The trimer, that is, a three-octapod chain, is the shortest stable chain of interlocked octapods. Two-octapod chains (dimers) are suppressed, which implies that they are unstable. Dynamic fluctuations and the incorporation of trimers and of longer oligomers are mostly responsible for the growth of the interlocked chains, which in this way can reach up to ∼20 octapods in length. Besides tracking nucleation and growth, the need for *in situ* observations is illustrated by measurements of important properties, such as the interparticle spacing in the chains. In their native solution environment, the octapod chains are much less densely packed than observed in conventional *ex situ* analysis after drying or vitrification of the solvent. On the basis of the characteristics obtained from *in situ* microscopy, a statistical mechanics model is built that explains the chain-length distribution (notably the absence of stable dimers), establishes the relative importance of van der Waals and entropic forces in the self-assembly, and determines the strength of the particle–particle interaction. Our findings illustrate the ability of *in situ* microscopy combined with modelling to obtain detailed quantitative insight into nanocrystal self-assembly processes in solution.

## Results

### *In situ* liquid-cell electron microscopy

**Figure 1:**
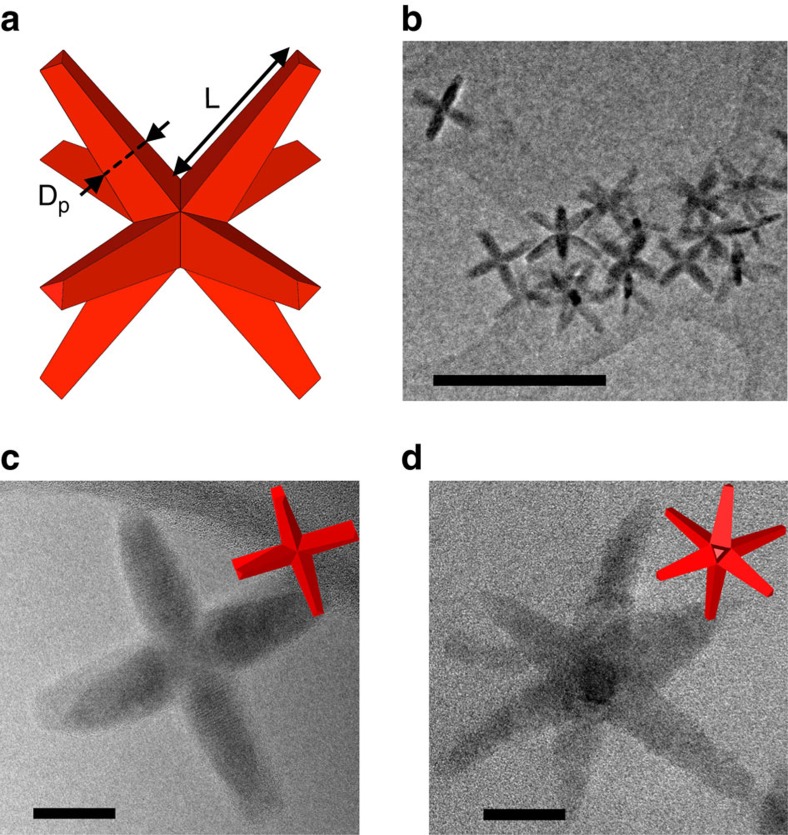
CdSe/CdS octapods. (**a**) Schematic representation of geometry of a single octapod-shaped CdSe/CdS nanocrystal. (**b**) TEM image of a group of CdSe/CdS octapods drop-cast onto amorphous carbon film. Scale bar, 200 nm. (**c**,**d**) High-magnification TEM images of octapods with different orientations. Scale bar, 20 nm. TEM, transmission electron microscopy.

*In situ* microscopy was performed by scanning transmission electron microscopy (STEM) using solutions of CdSe/CdS octapods (pod length *L*∼(47±3) nm, *L/D*_p_∼4.2; see Fig. 1) in toluene with a nominal particle concentration of 10^−8^ M, prepared according to reported methods^21^.

The solution was loaded in the liquid cell and observed within a viewing area where it was held between two electron transparent SiN_*x*_ membranes (see Methods). Immediately after starting the observations, there was a higher concentration of octapods near the corners of the cell (for example, Supplementary Figs 1 and 6) and along the edges of the window, whereas the centre of the cell remained less populated.

**Figure 2:**
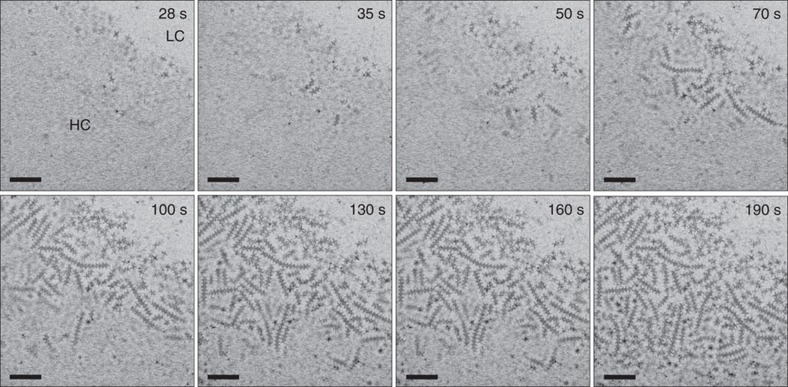
*In situ* LCEM of the assembly of octapods into linear chains. Selected inverted dark-field STEM images from Supplementary Movie 1 showing the formation of ordered chains of octapods in toluene. Scale bar, 500 nm. The images show the evolution of the system starting from single octapods dispersed in the entire liquid volume (for *t*<30 s, ‘HC'/‘LC': high/low concentration of octapods) to interlocked chain-like structures that aggregate near the top membrane of the liquid cell.

Figure 2 shows a representative sequence of liquid-cell STEM images of octapods in toluene (extracted from Supplementary Movie 1) that follows the time evolution near the boundary between high and low concentration. Initially (observation time *t*<30 s) the solution within these areas appears homogeneous, with diffuse contrast and streaking suggesting dispersed individual octapods and possibly chains submerged in the bulk solvent. As the self-assembly progresses, octapods and linear chains are clearly observed (*t*⩾35 s). Chains of interlocked octapods form exclusively in the region with higher concentration near the edges and corners of the viewing window (Supplementary Fig. 7). The first clear contrast is due to two short-chain segments, each containing three interlocked octapods (*t*=35 s). In the next ∼20 s, additional short chains become visible and the first longer chains appear; surrounding diffuse contrast indicates continuing rapid motion in the solvent. During the further evolution, the existing chains grow longer so that the field of view becomes progressively crowded with interlocked octapod chains of different length. An overall downward directionality might be interpreted as a sign of progressive drying of the solvent and concomitant increase of the octapod density in the remaining solution, such as in evaporative self-assembly processes^32^,^33^. Further examination shows, however, that the formation of octapod chains occurs in a 3D solution environment of constant thickness, and is not significantly influenced by evaporation of the toluene solvent (see Supplementary Note 1 and Supplementary Figs 3–6,8 and 9).

**Figure 3:**
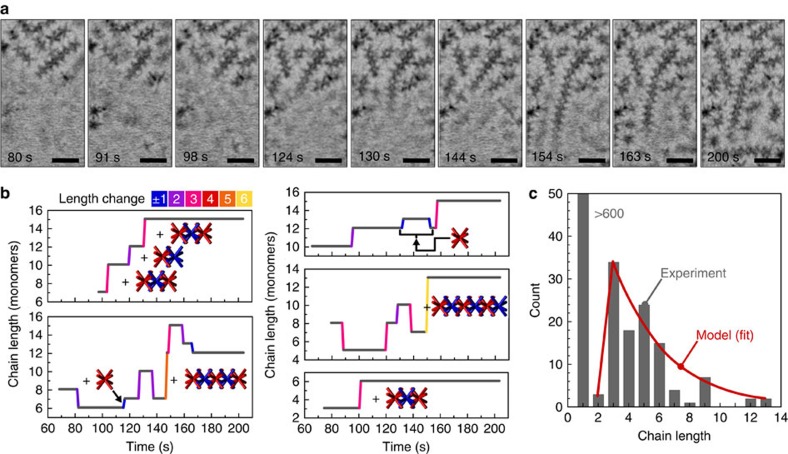
Imaging octapod chains and tracking their growth with monomer resolution; modelling of the assembly process. (**a**) Representative time-lapse series of inverted dark-field STEM images showing the growth of a long chain of interlocked octapods. Scale bar, 200 nm. (**b**) Representative traces of the chain length as a function of time, showing length changes occurring only in exceptional cases by addition or removal of monomers, but instead mostly via incorporation and detachment of longer oligomers. Length changes are colour coded as shown. Longer horizontal lines imply that the length stays unchanged for extended time periods. (**c**) Length distribution of interlocked octapod chains, determined by *ex situ* STEM after opening the liquid cell (grey bars; total sample size: 752 objects, of which 642 were individual octapods). Note the absence of interlocked dimers. Red line: best fit of the length distribution within the statistical mechanics model described in the text.

Analysis of real-time image sequences allows us to develop a detailed picture of the self-assembly of 1D octapod chains in solution. Important elements, such as image contrast, predominant octapod orientations and a limited 3D imaging capability in STEM are discussed in the Methods and Supplementary Fig. 2. Figure 2 and additional data (Supplementary Movies 2–6; extracted images in Fig. 3 and Supplementary Figs 10–14) document the assembly mechanism. The process is initiated by the emergence of short segments of tightly interlocked octapod chains. The majority of chains are built from interlocked units, similar to those analysed *ex situ* in vitrified solvent^21^, but observations of pairs or larger numbers of monomers interacting tip-to-tip and diffusing as a non-interlocked cluster (Supplementary Movies 2 and 3) suggest a richer set of interactions between monomers than the pod-to-pod van der Waals forces invoked so far.

**Figure 4:**
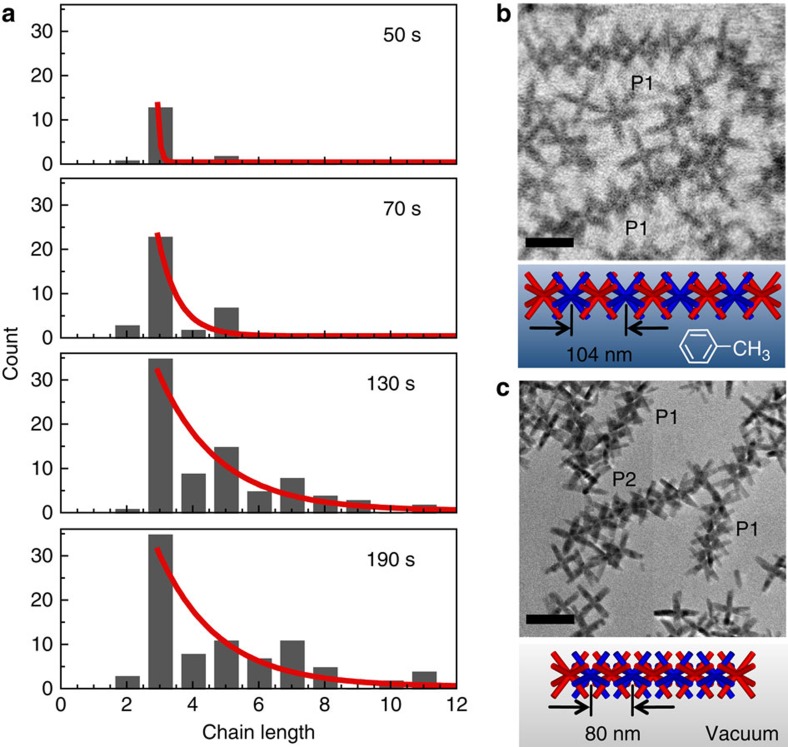
Characteristics of octapod chains determined *in situ*. (**a**) Length distribution at different times during the self-assembly. Red lines are exponential fits for chain lengths ⩾3. (**b**) Octapod chains in toluene, imaged *in situ* by liquid-cell STEM. Two longer chains are imaged in projection **P1** (see Methods and Supplementary Fig. 2). (**c**) Octapod chains in projections **P1** and **P2** imaged by TEM in vacuum after opening the liquid cell, shown at the same scale as **b**. Scale bars, 100 nm. Note the substantially larger interparticle spacing observed in toluene (52±2 nm) compared with the dried assemblies (40±2 nm), which suggests that capillary forces upon drying (and during freezing for cryo-TEM)^21^ cause a significant tightening of the chains. TEM, transmission electron microscopy.

Short interlocked octapod chains typically emerge from the bulk solution, which indicates that they are formed there. Observations of the fluid with focal plane near the top SiN_*x*_ membrane show the attachment and detachment of such short-chain segments as the predominant process of forming long 1D chains (Fig. 2). Chain growth is clearly a dynamic process, subject to fluctuations that add and remove segments of varying length (Fig. 3a,b). The smallest interlocked oligomer unit observed in our experiments is the trimer, that is, consists of three octapods. A survey of a large number of chains *ex situ* after opening the liquid cell (Fig. 3c) and *in situ* at different stages of the assembly (Fig. 4a) shows only isolated instances of interlocked dimers, whereas trimers and longer chains are much more abundant. The addition of trimers and longer oligomers is also the predominant elementary step in chain growth (Fig. 3b), while monomer incorporation is quite rare and is often followed by detachment of the same unit, suggesting incomplete locking (for example, Fig. 3b). Two-octapod increments are occasionally seen (Fig. 3b) but the absence of stable dimers in the solution suggests that these instances may actually represent a sequential addition of monomers or of non-interlocked dimers.

The observed solution-phase self-assembly deviates from a classic nucleation and growth scenario, which presumes the formation of a stable nucleus followed by attachment of monomer units^34^. While there appears to be a stable nucleus, the trimer, the further growth in most cases does not involve the incorporation of individual octapods, even though monomers are abundant and show extensive Brownian motion (Supplementary Note 2 and Supplementary Fig. 16). Instead, the stable nucleus grows primarily by attachment of other supercritical units, all of which are mobile in the solution. Attachment is facilitated by a considerable bending flexibility of the chains, which, for instance, allows long chains near the membrane to reach into the bulk liquid to capture a diffusing segment (Fig. 3a, Supplementary Fig. 10 and Supplementary Movie 4).

### Statistical mechanical model

We constructed a model to probe the physical origin of the striking suppression of dimers and the prevalence of trimers as the shortest chain of interlocked octapods in solution. Using the measured chain-length distribution and other characteristics identified uniquely by *in situ* microscopy, the model provides insight into the interactions underlying the self-assembly process. In equilibrium, the probability of finding a chain of length *n* can be determined from the grand canonical distribution function:


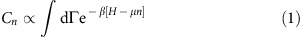


Here the chemical potential of octapods is determined by the monomer concentration, *C*_1_, as *μ*=*kT* In *C*_1_; *H* is the interaction Hamiltonian of the system, *β*=1/*kT* and dΓ represents an integration over internal degrees of freedom of the chain, that is, positions and orientations of *n*−1 octapods. If we neglect physical binding between the octapods (that is, assume *H*=0, but exclude their mutual overlap), the confinement of a single octapod within a cage made by two of its neighbours can be represented as a factor *v*. This factor has the meaning of an effective localization volume and it accounts for the loss of translational and orientational entropies of the confined octapod. If Δ is a typical range of translational fluctuations, the corresponding amplitude of the angular degrees of freedom is Δ/*R*, where *R* is a characteristic radius of the octapod. Therefore an integration over three translational and three orientational degrees of freedom gives an effective localization volume *v*∼Δ^6^/*R*^3^.

We can now take into account mutual binding of the octapods. In our model, two octapods in contact create reversible bonds, each having the same characteristic binding energy 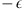 and localization range *δ* (∼2.5 nm, set by the length of the ligands). We represent these bonds as a collection of two-state systems that can be in a bound or an unbound state independently of each other. This leads to the following distribution function:


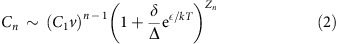


An alternative, more detailed derivation of this result is presented in Supplementary Note 3. *Z*_*n*_ is the total number of contacts between the octapods that can be independently formed within the chain. This parameter is of crucial importance for understanding the observed chain statistics, in particular the substantial suppression of dimers compared with longer chains. We assume the maximum number of contacts that can be made between nearest neighbours to be 4. This implies that a tighter interlocking of two octapods with eight mutual contacts is not sterically allowed, presumably because of the geometry of their octahedral cores. This is consistent with the experimental observations (see below). In addition to the interactions with its nearest neighbours, it is assumed that fluctuations in the chain allow an octapod to create up to four contacts with any of its next-nearest neighbours. This consideration sets an upper bound to the number of contacts in a chain: *Z*_*n*_≤4(*n*−1)+4(*n*−2)=8*n*−12. However, this is not the only limitation. Each octapod has six degrees of freedom: three translational and three rotational. This means that for trimers (*n*=3) the number of possible contacts (12) is exactly equal to the number of internal degrees of freedom of the chain, 6(*n*−1). For dimers (*n*=2), the number of contacts is less than the number of degrees of freedom (4 and 6, respectively), while for chains longer than (*n*⩾3), the situation is opposite. The maximally connected structure with *Z*_*n*_=8*n*−12 would be over-constrained, and can only exist if the shapes are identical and symmetric to a very high precision. Any imperfection would lead to a loss of some of the contacts. We therefore postulate that the number of internal degrees of freedom sets another bound on the value of *Z*_*n*_, that is,


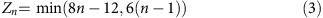


This model can successfully describe the observed chain statistics, including the suppression of dimers. In fact, its major predictions are very robust with respect to the choice of the model parameters. For *n*⩾3, *Z*_*n*_=6(*n*−1), that is, the overall distribution function is proportional to an exponential, *C*_*n*_∝*p*^*n*−1^, where 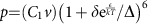. For *n*=2, *Z*_*n*_=4, that is, dimers are suppressed by a factor (*C*_1_*v*)^1/3^.

As far as the specific parameters are concerned, the characteristic radius *R* of the octapod is set to ∼30 nm, half the pod length; Δ≈15 nm was estimated as the difference between the apparent interparticle distance in the chain (50 nm) and its minimal value allowed geometrically (35 nm). The estimated average concentration of free octapods in the area in which self-assembly takes place is *C*_1_∼10^−6^ M∼10^−6^ nm^−3^, so that C_1_*ν*∼C_1_Δ^6^/*R*^3^ is of order 10^−3^.

## Discussion

Once the geometrical parameters are fixed, the only free parameter of the model is the energy per contact, 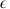. The value of the energy that gives the best fit to the experimental data is 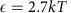 (Fig. 3c), approximately twice the energy expected for a pure van der Waals interaction between two crossed pods^21^. The difference may be due to effects of inter-digitation of the ligands. The robustness of our model can be illustrated by the fact that changing *C*_1_*v* by a factor of 2 leads to a correction to the binding strength 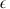 of 0.1*kT*, and to a relative change of the dimer fraction of only 25%. Additional molecular dynamics simulations also show that a pure van der Waals interaction is not sufficient to induce assembly (see Supplementary Note 4, Supplementary Methods and Supplementary Figs 17–19). Adding a short-range attraction does lead to chain formation.

In our model the assembly is driven not only by the energy gained because of binding. The fact that each contact fluctuates between bound and unbound states results in an additional entropic-free energy contribution 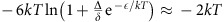 per particle that stabilizes the chain. A final piece of evidence that supports the proposed mechanism stems from measurements of the octapod spacing in chains, determined *in situ* to avoid uncertainties due to capillary forces that may affect *ex situ* measurements of the lattice constant of dried or vitrified assemblies. The observed next-nearest neighbour octapod distance in solution (≈100 nm, Fig. 4b) is in fact greater than the one needed for physical contact between four tips of the two octapods (≈85 nm, close to the value found *ex situ* in vacuum (Fig. 4c) or vitrified solvent^21^). This distance is however sufficient to make up to two simultaneous contacts upon some rotation. In this way, the system can achieve the required value of *Z*_*n*_ (6 per particle) without paying an excessive entropic penalty for the extra confinement. The loose packing in solution also rationalizes characteristics important to chain growth, such as the facile addition and removal of segments and the bending of chains in tight radii, involved in capturing chain segments at later growth stages.

By imaging the self-assembly of branched nanocrystals with monomer resolution in solution, our experiments have pointed out a novel avenue for studying key aspects of self-assembly, such as the mechanism of formation and growth of ordered nanocrystal arrays and the interparticle spacing in solution. On the basis of characteristics obtained from *in situ* microscopy, a statistical mechanics model could be built that established the relative importance of van der Waals and entropic forces and determined the strength of the particle–particle interaction. A similar approach can be applied to other systems, such as spherical or polyhedral particles that arrange in two-dimensional or 3D assemblies, binary or more complex nanocrystal mixtures, or the folding and assembly of suitably tagged proteins. Once cells are developed that allow for controlled mixing of different liquids, external stimuli (for example, changes in PH, addition of co-solvents or depletants and so on.) may be used to actively change the assembly process while observing the outcome in real time.

## Methods

### Materials preparation

CdSe/CdS octapod-shaped nanocrystals were synthesized following a procedure based on the growth of CdS pods on cuboctahedral CdSe seeds that are prepared after a cation exchange process from Cu_2−*x*_Se nanocrystals. Briefly, a reaction flask was loaded with 120 mg of CdO and various surfactants (6 g of trioctylphosphine oxide, 160 mg of hexylphosphonic acid and 580 mg of octadecylphosphonic acid). The flask was kept under vacuum at 150 °C for 120 min. The temperature was increased to 350 °C and 1.6 g of trioctylphosphine was injected. After the recovery of the temperature, an aliquot of previously prepared Cu_2−*x*_Se nanocrystals with a concentration of 0.3 × 10^−9^ M was quickly injected in the flask after the addition of 16:0.5 mg S/trioctylphosphine solution. The reaction was run for 10 min and cooled down to the room temperature. After several washing steps with an anti-solvent, the final product was dispersed in 5 ml of toluene.

### *In situ* liquid-cell STEM experiments

The liquid-cell experiments were carried out in a dedicated specimen holder (Hummingbird Scientific) using micro-fabricated liquid cells, consisting of two 30-nm-thick SiN_*x*_ membrane windows with 50 μm × 50 μm window area supported by Si frames. The spacing between the windows was controlled using 200 and 300 nm polystyrene beads or 100 and 200 nm SiO_2_ spacers. STEM imaging was performed in a FEI Titan 80–300 environmental Cs image corrected microscope operated at 300 kV. Low-loss electron energy loss spectra (EELS) were acquired using a Gatan Enfina spectrometer with an entrance aperture semi-angle of 5 mrad. The weighted average of the ratio of the thickness and the inelastic mean free path for the toluene and the two SiN membranes (each 30 nm thick) was determined from low-loss EELS, and the toluene thickness was calculated from it according to ref. 35 (Supplementary Figs 3,5 and 6). Typically, ∼1 μl of the octapod-containing solution with an initial concentration of particles of 10^−8^ M was introduced in the liquid cell. The holder was inserted in the microscope immediately after loading of the cell. The time delay between loading of the solution and initiating the observations was ∼10–20 min. STEM imaging was performed with ∼2 Å probe size and∼0.2–0.3 nA beam current, measured in vacuum before introduction of the liquid cell. Time-lapse series of STEM images were acquired at several positions along the cell window, and at all positions the assembly of octapods was observed in real time. The results of the observations at two different areas are shown in Supplementary Movies 1 and 8. For Supplementary Movies 1–7 time-lapse STEM images of the assembly of the octapods were recorded with frame interval of 1 s and 3.96 μs dwell time/pixel. The conditions for the acquisition of STEM images were: electron dose rate of 1.35 e^−^ Å^−2^ s^−1^, electron dose per image of 1.35 e^−^ Å^−2^, total electron dose for the movie (204 frames) of 275.4 e^−^ Å^−2^. The Supplementary Movies 1–7 are played at 20 frames per second. The original STEM images are shown in Supplementary Fig. 1. The STEM images in which the octapods appear dark on a light background have been inverted (in the program ImageJ) to facilitate visual perception and analysis. Supplementary Movie 8 is a series of time-lapse images recorded at a frame interval of 9 s.

### Image contrast in *in situ* liquid STEM of octapod assemblies

The use of a focused probe in STEM, whose focal plane coincides with one of the membranes (for example, the top membrane) of the liquid cell, implies a limited 3D imaging capability. As an octapod chain transits from the bulk solution towards the membrane (Supplementary Fig. 2a, top), it initially appears with soft contrast that progressively sharpens (Supplementary Fig. 2a, bottom). Electron beam exposure generally induces octapods and chains to move from the interior of the solution towards the SiN_*x*_ membrane, possibly as a result of a beam-induced charging of the membrane and interaction with the tips of the polar (wurtzite) CdS pods via Coulomb or higher order multipole forces. While this behaviour facilitates high-resolution imaging by concentrating material near the focal plane, which allows to clearly distinguish individual octapods and their orientation in solution and within 1D chains, the attraction to the membrane is sufficiently weak that free octapod monomers can move via Brownian motion in the solution (see Supplementary Note 2 and Supplementary Fig. 16) with a substantially larger diffusion coefficient (*D*_t_∼10^−15^ m^2^ s^−1^) than found previously for particles in ultrathin liquid layers^36^. Octapod chains also show significant freedom of motion, including in-plane and out-of plane bending (Supplementary Fig. 10) and diffusion (Supplementary Fig. 11).

The interaction with the SiN_*x*_ membrane also acts to reduce the phase space of possible orientations of octapod chains (Supplementary Figs 2d,e and 15), which aids in the analysis of the chain length and allows counting the number of octapods in interlocked 1D chains with monomer resolution. Surveying a large number of chains, a large majority appears in projection **P1**, in which the pods of neighbouring nanoparticles project symmetrically towards the membrane. A minority of chains shows projection **P2**, in which they are rotated by 22.5° around their axis relative to **P1**. Occasionally, octapod chains with mixed appearance (that is, partial **P1** and **P2**) are observed. While still interlocked, these chains contain a stacking defect in which one section (a four-octapod segment in orientation **P2**, in Supplementary Fig. 2e) is rotated by 22.5° relative to the remainder of the chain (a five-octapod segment in projection **P1**, Supplementary Fig. 2e).

## Additional information

**How to cite this article**: Sutter, E. *et al*. *In-situ* microscopy of the self-assembly of branched nanocrystals in solution. *Nat. Commun.* 7:11213 doi: 10.1038/ncomms11213 (2016).

## Supplementary Material

Supplementary Information Supplementary Figures 1-19, Supplementary Notes 1-4, Supplementary Methods and Supplementary References

Supplementary Movie 1 Time-lapse sequence #1 of liquid cell STEM images following the self-assembly of branched octapod nanocrystals in toluene.

Supplementary Movie 2 Time-lapse sequence #2 of liquid cell STEM images following the self-assembly of branched octapod nanocrystals in toluene

Supplementary Movie 3 Time-lapse sequence #3 of liquid cell STEM images following the self-assembly of branched octapod nanocrystals in toluene.

Supplementary Movie 4 Time-lapse sequence #4 of liquid cell STEM images following the self-assembly of branched octapod nanocrystals in toluene.

Supplementary Movie 5 Time-lapse sequence #5 of liquid cell STEM images following the self-assembly of branched octapod nanocrystals in toluene

Supplementary Movie 6 Time-lapse sequence #6 of liquid cell STEM images following the self-assembly of branched octapod nanocrystals in toluene.

Supplementary Movie 7 Time-lapse sequence #7 of liquid cell STEM images following the self-assembly of branched octapod nanocrystals in toluene.

Supplementary Movie 8 Time-lapse sequence #8 of liquid cell STEM images following the self-assembly of branched octapod nanocrystals in toluene.

## References

[b1] Konstantatos G., Clifford J., Levina L. & Sargent E. H. Sensitive solution-processed visible-wavelength photodetectors. Nat. Photon. 1, 531–534 (2007) .

[b2] Sun L. . Bright infrared quantum-dot light-emitting diodes through inter-dot spacing control. Nat. Nanotechnol. 7, 369–373 (2012) . 22562037 10.1038/nnano.2012.63

[b3] Tao A., Sinsermsuksakul P. & Yang P. Tunable plasmonic lattices of silver nanocrystals. Nat. Nanotechnol. 2, 435–440 (2007) . 18654329 10.1038/nnano.2007.189

[b4] Dong A., Jiao Y. & Milliron D. J. Electronically coupled nanocrystal superlattice films by *in situ* ligand exchange at the liquid–air interface. ACS Nano 7, 10978–10984 (2013) . 24252075 10.1021/nn404566b

[b5] Choi J.-H. . Bandlike transport in strongly coupled and doped quantum dot solids: a route to high-performance thin-film electronics. Nano Lett. 12, 2631–2638 (2012) . 22509936 10.1021/nl301104z

[b6] Cheon J. . Magnetic superlattices and their nanoscale phase transition effects. Proc. Natl Acad. Sci. USA 103, 3023–3027 (2006) . 16492783 10.1073/pnas.0508877103 PMC1413888

[b7] Yamada Y. . Nanocrystal bilayer for tandem catalysis. Nat. Chem. 3, 372–376 (2011) . 21505495 10.1038/nchem.1018

[b8] Kim Y., Shah A. A. & Solomon M. J. Spatially and temporally reconfigurable assembly of colloidal crystals. Nat. Commun. 5, 3676 (2014) . 24759549 10.1038/ncomms4676

[b9] Collier C. P., Vossmeyer T. & Heath J. R. Nanocrystal superlattices. Annu. Rev. Phys. Chem. 49, 371–404 (1998) . 15012432 10.1146/annurev.physchem.49.1.371

[b10] Shevchenko E. V., Talapin D. V., Kotov N. A., O'Brien S. & Murray C. B. Structural diversity in binary nanoparticle superlattices. Nature 439, 55–59 (2006) . 16397494 10.1038/nature04414

[b11] Frenkel D. Entropy-driven phase transitions. Phys. A Stat. Mech. Appl. 263, 26–38 (1999) .

[b12] Mirkin C. A., Letsinger R. L., Mucic R. C. & Storhoff J. J. A DNA-based method for rationally assembling nanoparticles into macroscopic materials. Nature 382, 607–609 (1996) . 8757129 10.1038/382607a0

[b13] Park S. Y. . DNA-programmable nanoparticle crystallization. Nature 451, 553–556 (2008) . 18235497 10.1038/nature06508

[b14] Nykypanchuk D., Maye M. M., van der Lelie D. & Gang O. DNA-guided crystallization of colloidal nanoparticles. Nature 451, 549–552 (2008) . 18235496 10.1038/nature06560

[b15] Damasceno P. F., Engel M. & Glotzer S. C. Predictive self-assembly of polyhedra into complex structures. Science 337, 453–457 (2012) . 22837525 10.1126/science.1220869

[b16] Auer S. & Frenkel D. Prediction of absolute crystal-nucleation rate in hard-sphere colloids. Nature 409, 1020–1023 (2001) . 11234006 10.1038/35059035

[b17] Cacciuto A., Auer S. & Frenkel D. Onset of heterogeneous crystal nucleation in colloidal suspensions. Nature 428, 404–406 (2004) . 15042084 10.1038/nature02397

[b18] Gasser U., Weeks E. R., Schofield A., Pusey P. N. & Weitz D. A. Real-space imaging of nucleation and growth in colloidal crystallization. Science 292, 258–262 (2001) . 11303095 10.1126/science.1058457

[b19] Yethiraj A. & van Blaaderen A. A colloidal model system with an interaction tunable from hard sphere to soft and dipolar. Nature 421, 513–517 (2003) . 12556887 10.1038/nature01328

[b20] Vutukuri H. R., Imhof A. & van Blaaderen A. Fabrication of polyhedral particles from spherical colloids and their self-assembly into rotator phases. Angew Chem. Int. Ed. Engl. 53, 13830–13834 (2014) . 25366869 10.1002/anie.201409594 PMC4502970

[b21] Miszta K. . Hierarchical self-assembly of suspended branched colloidal nanocrystals into superlattice structures. Nat. Mater. 10, 872–876 (2011) . 21946613 10.1038/nmat3121

[b22] Williamson M. J., Tromp R. M., Vereecken P. M., Hull R. & Ross F. M. Dynamic microscopy of nanoscale cluster growth at the solid-liquid interface. Nat. Mater. 2, 532–536 (2003) . 12872162 10.1038/nmat944

[b23] Zheng H. M. . Observation of single colloidal platinum nanocrystal growth trajectories. Science 324, 1309–1312 (2009) . 19498166 10.1126/science.1172104

[b24] Sutter E. . *In situ* liquid cell electron microscopy of Ag-Pd galvanic replacement reactions on Ag nanoparticles. Nat. Commun. 5, 4946 (2014) . 25208691 10.1038/ncomms5946

[b25] Park J. . Direct observation of nanoparticle superlattice formation by using liquid cell transmission electron microscopy. ACS Nano 6, 2078–2085 (2012) . 22360715 10.1021/nn203837m

[b26] Rogers W. B. & Manoharan V. N. Programming colloidal phase transitions with DNA strand displacement. Science 347, 639–642 (2015) . 25657244 10.1126/science.1259762

[b27] Maye M. M. . Switching binary states of nanoparticle superlattices and dimer clusters by DNA strands. Nat. Nanotechnol. 5, 116–120 (2010) . 20023646 10.1038/nnano.2009.378

[b28] Li H., Kanaras A. G. & Manna L. Colloidal branched semiconductor nanocrystals: state of the art and perspectives. Acc. Chem. Res. 46, 1387–1396 (2013) . 23369428 10.1021/ar3002409

[b29] Glotzer S. C. & Solomon M. J. Anisotropy of building blocks and their assembly into complex structures. Nat. Mater. 6, 557–562 (2007) . 17667968 10.1038/nmat1949

[b30] Conca E. . Charge separation in Pt-decorated CdSe@CdS octapod nanocrystals. Nanoscale 6, 2238–2243 (2014) . 24424255 10.1039/c3nr05567a

[b31] Qi W. . Ordered two-dimensional superstructures of colloidal octapod-shaped nanocrystals on flat substrates. Nano Lett. 12, 5299–5303 (2012) . 22938387 10.1021/nl302620j

[b32] Brinker C. J., Lu Y., Sellinger A. & Fan H. Evaporation-induced self-assembly: nanostructures made easy. Adv. Mater. 11, 579–585 (1999) .

[b33] Rabani E., Reichman D. R., Geissler P. L. & Brus L. E. Drying-mediated self-assembly of nanoparticles. Nature 426, 271–274 (2003) . 14628047 10.1038/nature02087

[b34] De Yoreo J. J., Vekilov P. in Biomineralization eds Dove P.M., De Yoreo J.J., Weiner S. 57–93Mineralogical Society of America (2003) .

[b35] Egerton R. F. Electron Energy Loss Spectroscopy in the Electron Microscope Plenum Press (1996) .

[b36] Lu J. Y., Aabdin Z., Loh N. D., Bhattacharya D. & Mirsaidov U. Nanoparticle dynamics in a nanodroplet. Nano Lett. 14, 2111–2115 (2014) . 24641092 10.1021/nl500766j

