# Supplementary material for: In situ microscopy of the self-assembly of branched nanocrystals in solution
Source: Nat Commun. 2016 Apr 4;7:11213. doi: 10.1038/ncomms11213 (PMC4822026; doi:10.1038/ncomms11213)
Supplement: Supplementary Information — Supplementary Figures 1-19, Supplementary Notes 1-4, Supplementary Methods and Supplementary References [file ncomms11213-s1.pdf]

Supplementary Figures

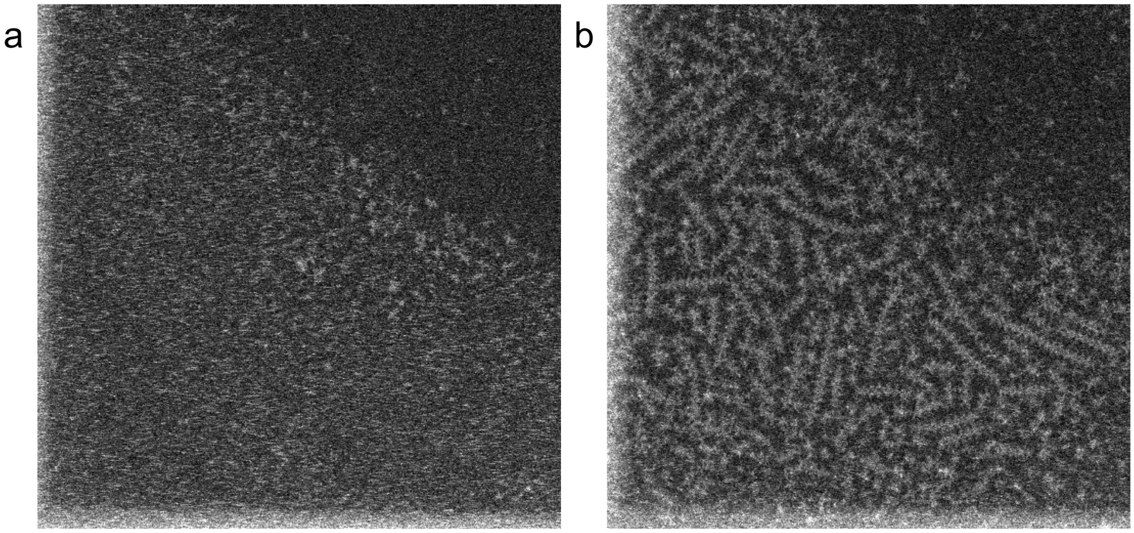

Supplementary Figure 1. Unprocessed scanning transmission electron microscopy (STEM) images of octapod self-assembly into linear chains. Unprocessed dark-field STEM images at **a.** t =1 s and **b.** t = 204 s from supplementary movie 1 acquired at the corner of the window of the liquid cell (brighter lines left and bottom). FOV: 3728 nm × 3728 nm. The initial image (a) shows contrast variation related to the distribution of octapods in the cell about 30 minutes after loading of the toluene-octapod solution in the cell. The contrast variation corresponds to a transition between an area (top, right) with lower density of octapods to an area with higher density of octapods where most of the assembly into chains is observed.

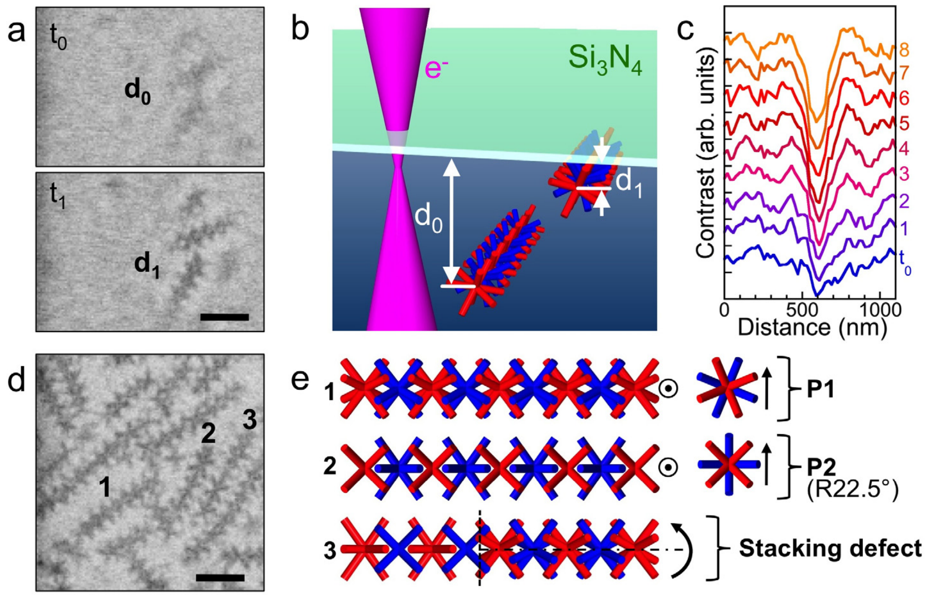

Supplementary Figure 2. Image contrast in in-situ liquid STEM of octapods assemblies. a. Time-lapse series of inverted dark-field STEM images obtained on the liquid cell, showing the same chains of interlocked octapods imaged submerged in the bulk toluene solvent (t_0), and aggregated near the top SiN_x membrane of the liquid cell ($t_1 = t_0 + 5$ s). Scale bar: 200 nm. **b.** Schematic of the situations shown in a., assuming a focal plane of the electron beam near the top SiN_x membrane. **c.** Time-dependent line profiles across one of the octapod chains shown in a. and b., illustrating the contrast change with change in depth. Numbers: elapsed observation time (s). **d.** Inverted dark-field STEM image showing several different configurations of octapod chains. Scale bar: 200 nm. **e.** Schematic top views (left) and frontal views (right) of the configurations shown in d. Cases 1 and 2 represent different projections, **P1** and **P2**, of defect-free interlocked chains. Arrow symbols indicate the direction of the electron beam. Case 3 refers to an interlocked chain containing a stacking defect, in which a segment of interlocked octapods is rotated by 22.5º relative to the remainder of the chain.

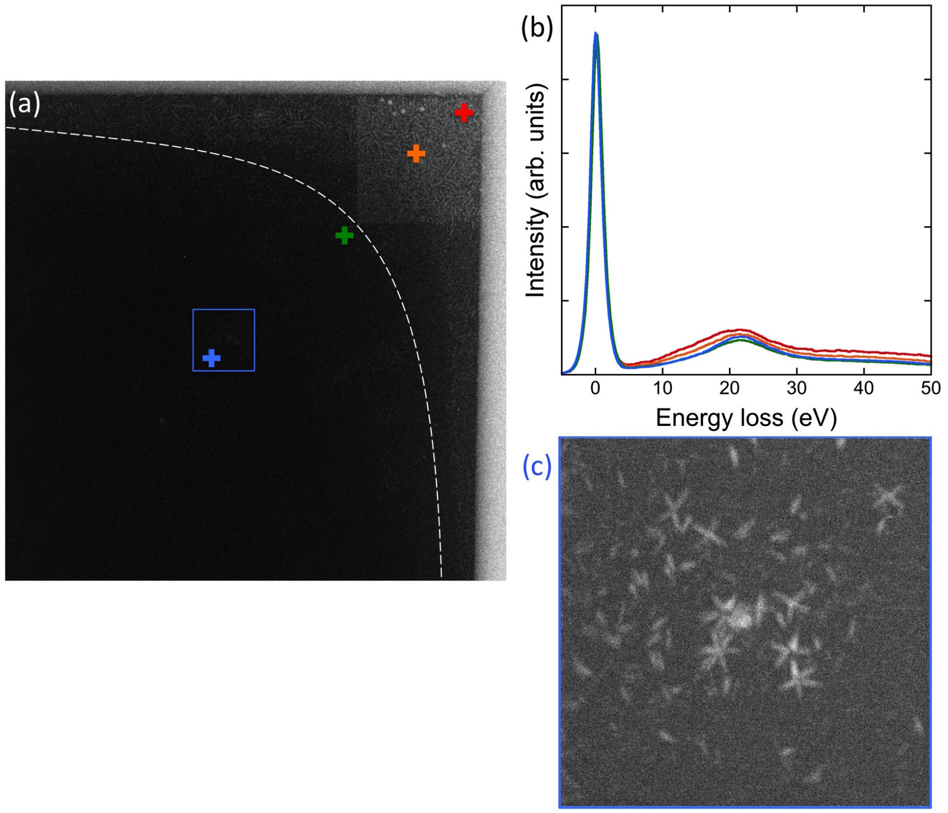

Supplementary Figure 3. STEM and STEM-EELS of the liquid cell with octapod chains in toluene. a. Low-magnification HAADF-STEM image of one corner of the window of the liquid cell (bright lines, top and right) loaded with octapods. Field of view (FOV): 15 μm × 15 μm; the entire window is 50 μm × 50 μm. The image was taken ~15 min. after starting the observations (and ~30 min. after loading) of the toluene-octapod solution in the liquid cell. It shows that the density of octapods is highest at the corner of the cell and along the edges of the cell window (to the right of and above the dashed line, high-concentration (HC) region). The image was taken after in-situ microscopy of the assembly of the octapods at the corner. Assembly of octapod chains is observed predominantly in the area of imaging (i.e., scanning of the electron beam). Outside the scan area, octapod chains are observed close to the area of high residual time of the electron beam (left edge of the FOV). **b.** Low-loss electron energy loss (EELS) spectra acquired at different points of the viewing window, marked in (a) with crosses of the same color. Measured thicknesses (from EELS spectra): 177 nm (red); 138 nm (orange); 110 nm (green); 119 nm (blue). **c.** Higher magnification HAADF STEM image of the area from the low-concentration (LC) region, showing the presence of mobile octapods and pods. The low-loss EELS spectrum acquired at this region (blue curve in (b)) indicates very similar thickness to the one closer to the corner of the cell. FOV: 1.328 μm × 1.328 μm.

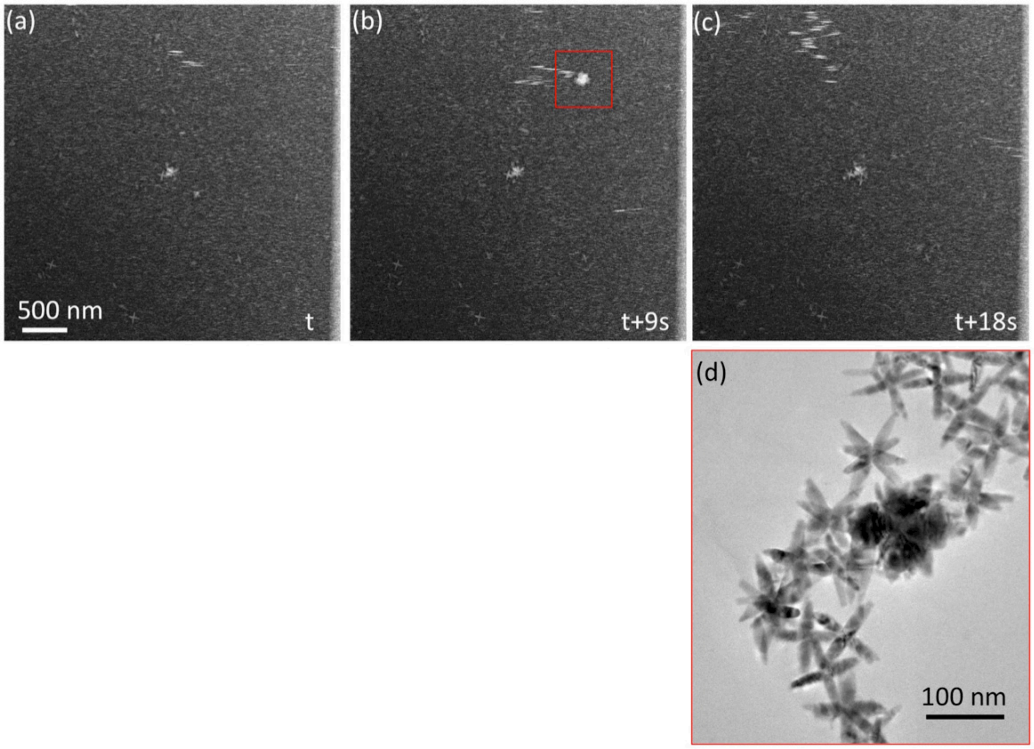

Supplementary Figure 4. STEM and STEM-EELS of the liquid cell with octapod chains in toluene. a. – c. Sequence of HAADF-STEM images (recorded at 9 s intervals) of the corner of the electron transparent window of the liquid cell shown in figure 1 (a). These images are part of the time-lapse sequence following the assembly of octapod chains in this particular area of the liquid cell (Supplementary Movie 8). The images show movement of octapods in this area. In particular, a large particle (bright) can be clearly distinguished in image (b). This particle is not present in the FOV initially (at time t, panel (a)) and has moved out of the FOV 9 s later (panel (c)). **d.** High–resolution TEM image of a particle similar to that observed in (b), shown among ordinary octapods. The size of these particles, which constitute a small percentage of nuclei that did not grow into octapods, is ~120 nm. The uninhibited movement of particles of such size confirms that the liquid thickness is >120 nm, consistent with the thickness calculated from low-loss EELS (Supplementary Fig. 3).

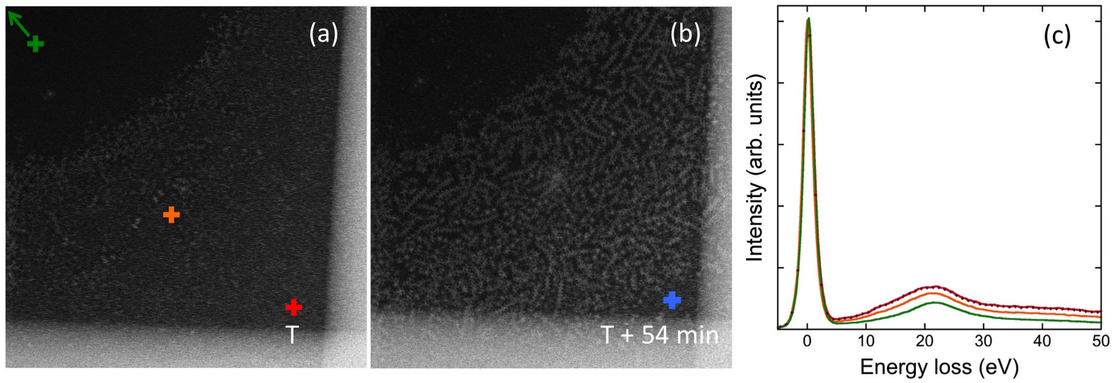

Supplementary Figure 5. STEM and STEM-EELS of the liquid cell with octapod chains in toluene. a. Low-magnification HAADF-STEM image of the corner of the window of the liquid cell (bright lines, left and bottom) ~ 15 min. after being loaded with octapods (FOV: 5.313 μm × 5.313 μm). **b.** The same area ~ 54 min. after the observation of self-assembly of the octapods in chains. **c.** Low-loss electron energy loss spectra acquired at different areas, marked with crosses of the same color in (a). Measured thicknesses (from EELS spectra): 190 nm (red); 138 nm (orange); 93 nm (green); 190 nm (blue). The blue and red spectra are taken at this location with a time interval of 54 min. confirming that the liquid layer thickness remained unchanged over the period of observations of the self-assembly of the octapods in chains. The green spectrum was obtained along the diagonal outside the field of view.

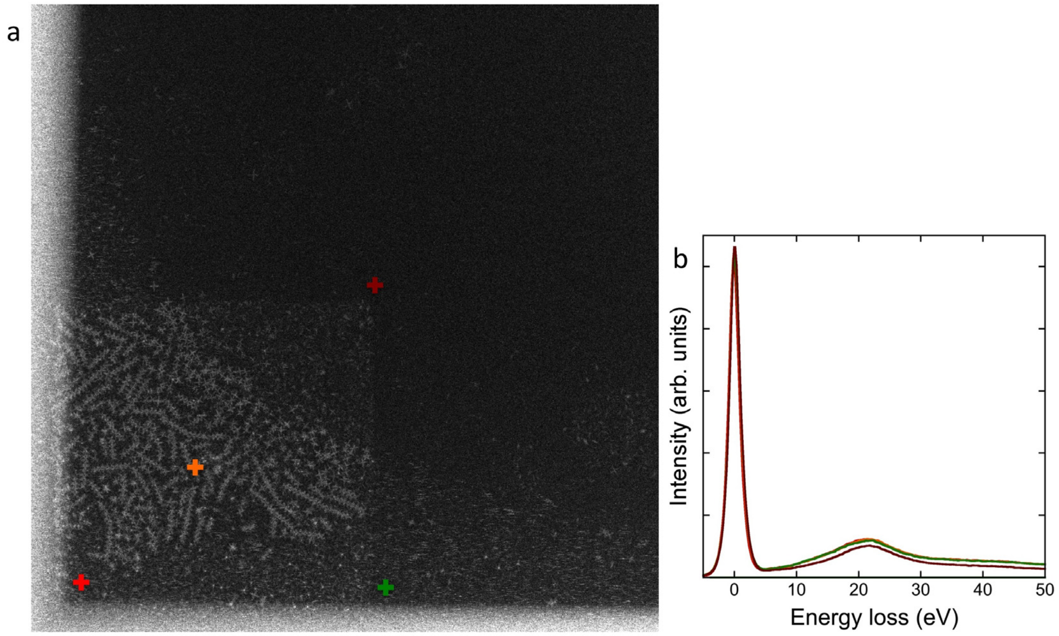

Supplementary Figure 6. STEM and STEM-EELS of the liquid cell with octapod chains in toluene. a. Low-magnification HAADF-STEM image of the corner of the window of the liquid cell (bright lines, left and bottom) loaded with octapods, whose assembly is followed in real time in movie M1 (FOV: 7.59 μm × 7.59 μm). **b.** Low-loss electron energy loss spectra acquired at different areas, marked with crosses of the same color in (a). Fluid thicknesses determined from the EELS spectra: 138 nm (red); 143 nm (orange); 143 nm (green); 105 nm (dark red).

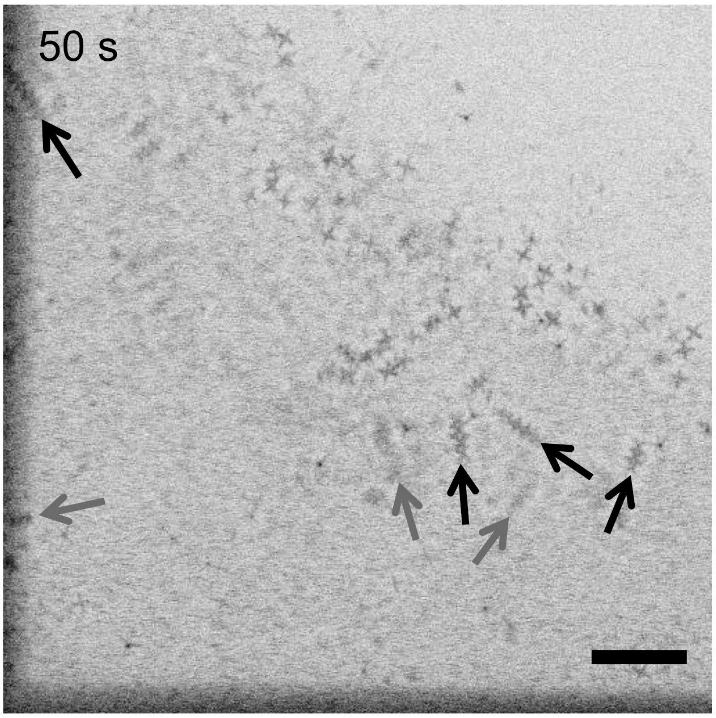

Supplementary Figure 7. Locations of the first longer linear octapod chains. Contrast inverted dark-field STEM image, extracted from supplementary movie 1, for time t = 50 s. First fully formed, linear chains of interlocked octapod are observed near the center of the field of view, as well as at the edge of the electron transparent SiN_x window (darker area at left and bottom). Black arrows: chains close to the SiN_x membrane (in focus). Gray arrows: submerged chains (out of focus). Scale bar: 500 nm.

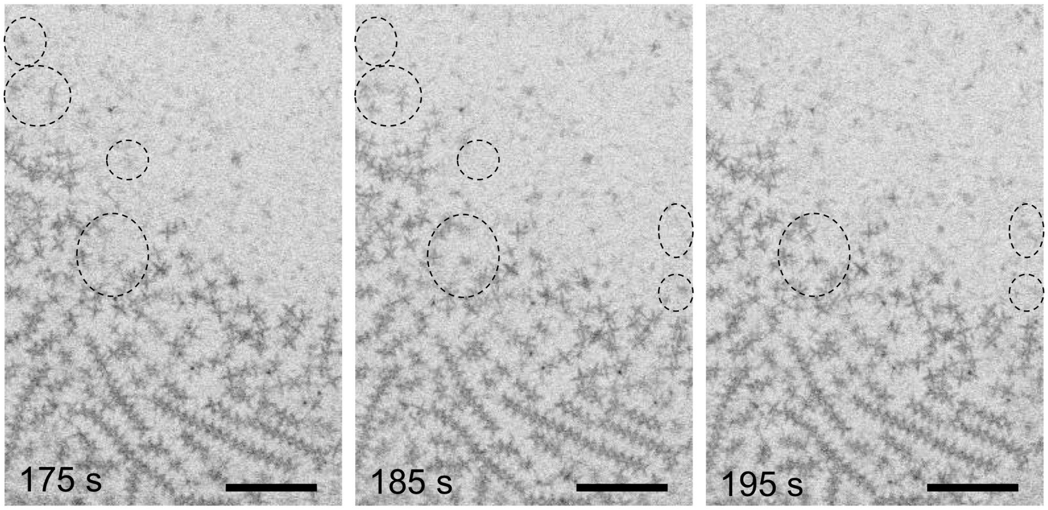

Supplementary Figure 8. Inverted dark-field STEM images showing displacement of octapods at later stages of the assembly process (for full time-lapse sequence, see Supplementary Movie 7). Dashed outlines mark areas that show changes in the distribution of objects in solution, thus providing evidence for the presence of solvent throughout the field of view at all stages of the assembly process. Scale bars: 500 nm.

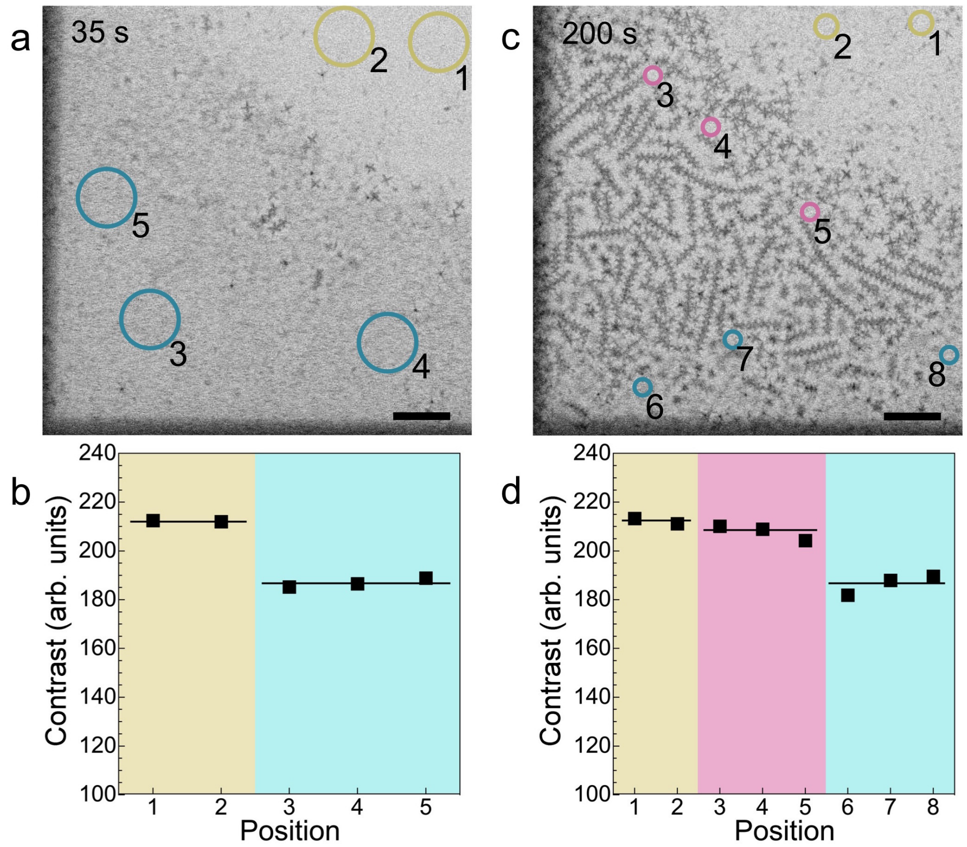

Supplementary Figure 9. Contrast at the early and late stages of octapod chain formation. a. Contrast-inverted dark-field STEM image, extracted from supplementary movie 1, for time t = 35 s. Scale bar: 500 nm. **b.** Mean image contrast measured at 5 positions marked in a. Positions (1) and (2) lie within the region with low octapod concentration; positions (3) to (5) are within the high-concentration region. Note that lower contrast corresponds to more electron scattering in the inverted dark-field STEM image. The analysis confirms the visual impression of darker contrast within the high-concentration area near the corner of the viewing window. **c.** Inverted dark-field STEM image, extracted from supplementary movie 1, for time t = 200 s. Scale bar: 500 nm. **d.** Image contrast measured at 8 positions marked in c. Positions (1) and (2) lie within the original region with low octapod concentration; positions (6) to (8) are within the remaining high-concentration region at the lower edge of the viewing window; positions (3) to (5) are in areas between longer octapod chains. Note that the STEM contrast at these positions is nearly identical to that in the low-concentration region.

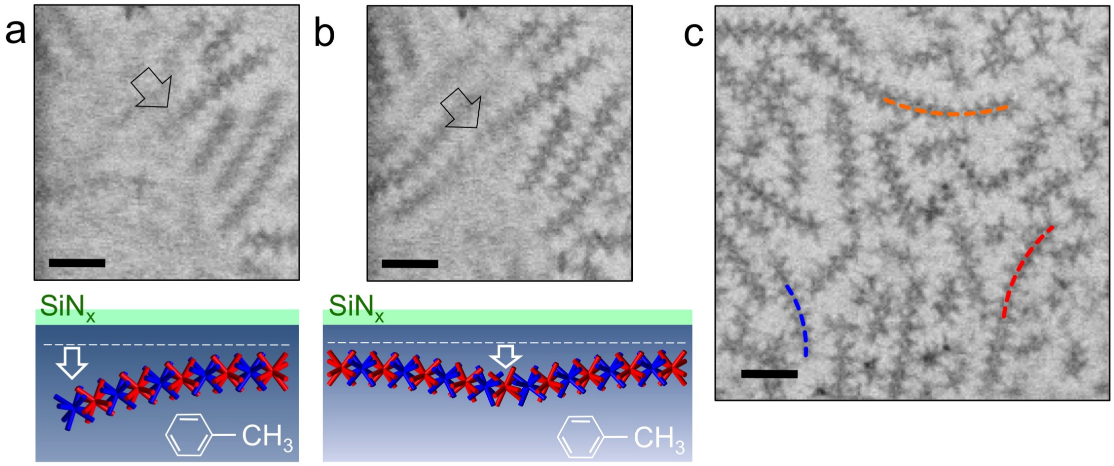

Supplementary Figure 10. Bending flexibility of interlocked 1D octapod chains. a., b. Out-of-plane bending of octapod chains located primarily near the SiN_x membrane of the liquid cell. Scale bars: 200 nm. **a.** Bending of one end of the chain into the bulk toluene solution, a process that facilitates the capturing of short chain segments that assemble in the 3D liquid environment. **b.** Downward bending near the center of an octapod chain, demonstrating the relatively weak interaction of the chain with the membrane. **c.** Bending of chains in the plane of the membrane, which is observed to change as a function of time, demonstrating the freedom of the 1D chains to change conformation in the plane. Measured bending radii are: 482 nm (blue), 608 nm (red), and 718 nm (orange).

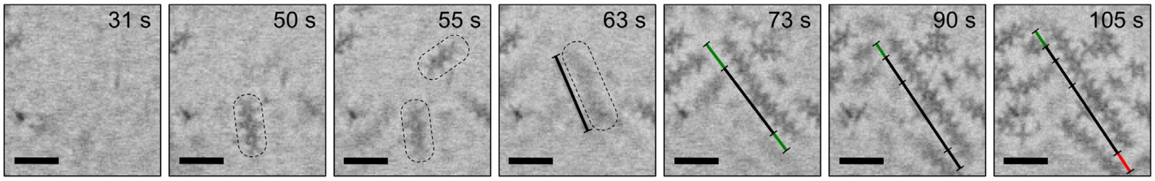

Supplementary Figure 11. Octapod chain behavior in solution (Detail from Supplementary Movie 3) Two short octapod segments (55 s) join in the solution to form a longer 1D chain (63 s). The further observation shows both growth (green) and shrinkage (red) by attachment and detachment of short chain segments. Scale bars: 200 nm.

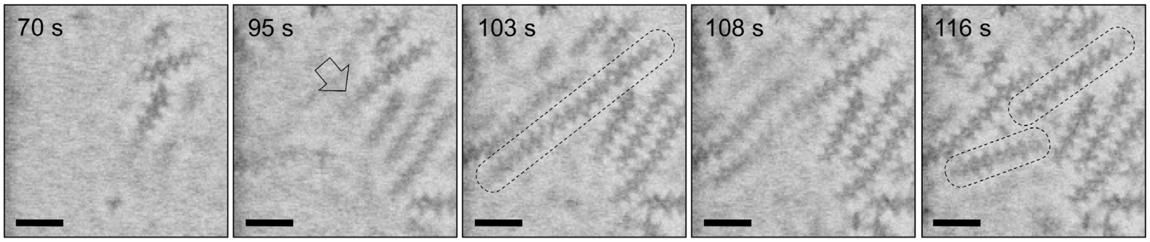

Supplementary Figure 12. Octapod chain behavior in solution (Detail from Supplementary Movie 4) A mis-rotated long octapod chain forms by combination of two segments (arrow). Dashed outlines: mis-rotated long chain (103 s); Separate fragments after detachment (116 s). Note the clearly visible exclusion zone around chains at 70 s, which delineates the area in which the local concentration of free octapods in the solution is reduced due to steric effects. Scale bars: 200 nm.

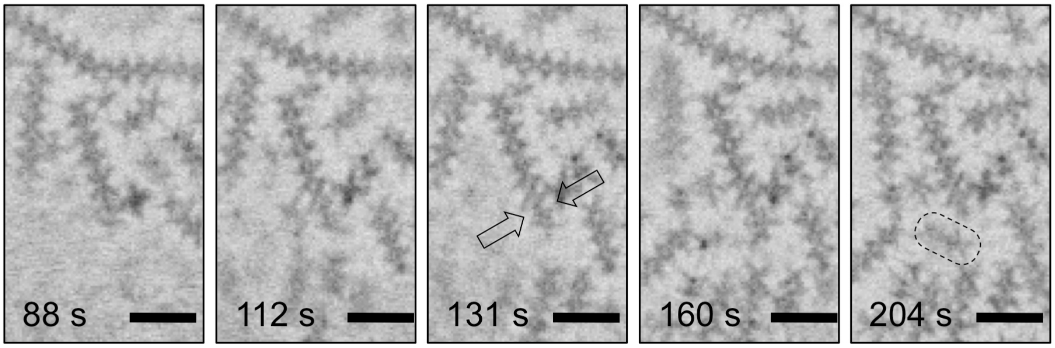

Supplementary Figure 13. Octapod chain behavior in solution (Detail from Supplementary Movie 5) A mis-rotated three-octapod segment attaches to the 1D chain in the center of the image (131 s, arrows), resides there (160 s), and eventually detaches (204 s). Scale bars: 200 nm.

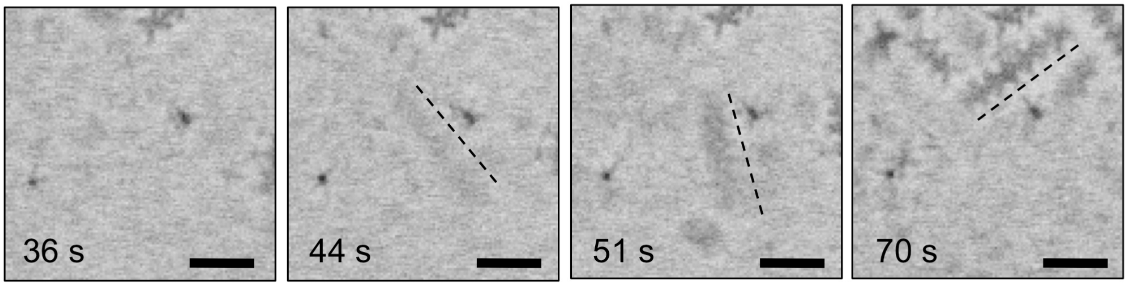

Supplementary Figure 14. Octapod chain behavior in solution (Detail from Supplementary Movie 6) A seven-octapod chain forms in the depth of the solution (44 s), moves toward the SiN_x membrane (51 s), and finally comes into focus near the membrane (70 s). Dashed lines indicate no change in the length of the 1D chain during this process. Scale bars: 200 nm.

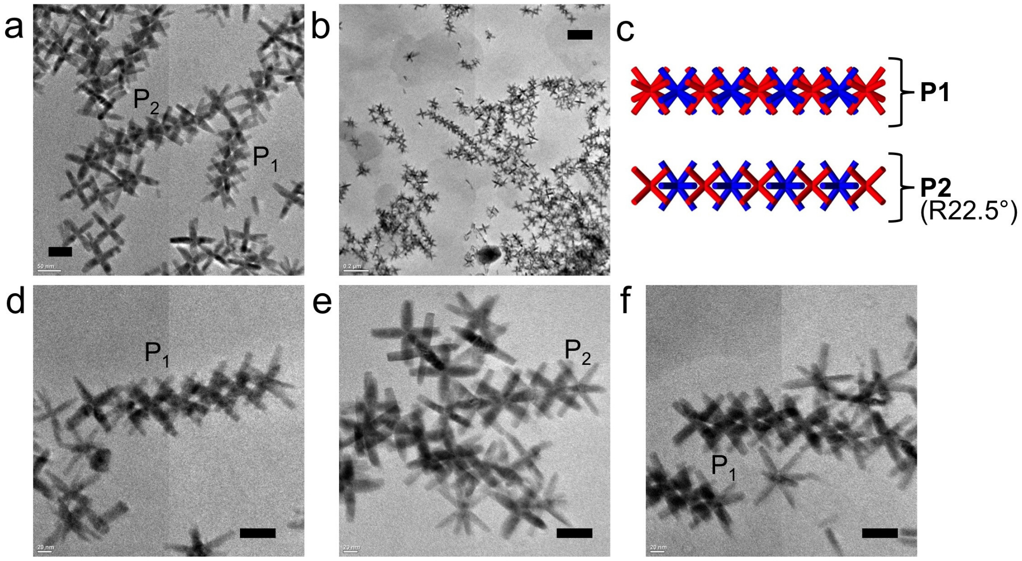

Supplementary Figure 15. Ex-situ TEM analysis of octapod chains after the in-situ experiment. a. Chains of octapods with two different orientations, **P1** and **P2**, supported by the SiN_x membrane after the disassembly of the liquid cell and the evaporation of the toluene. TEM image. Scale bar: 50 nm. **b.** Overview image of octapod assemblies. Scale bar: 200 nm. **c.** Schematic rendering of the two different orientations (**P1** and **P2**) of octapod chains. **d. – f.** Higher magnification TEM images of dry octapod chains on the SiN_x membrane. Scale bars: 50 nm.

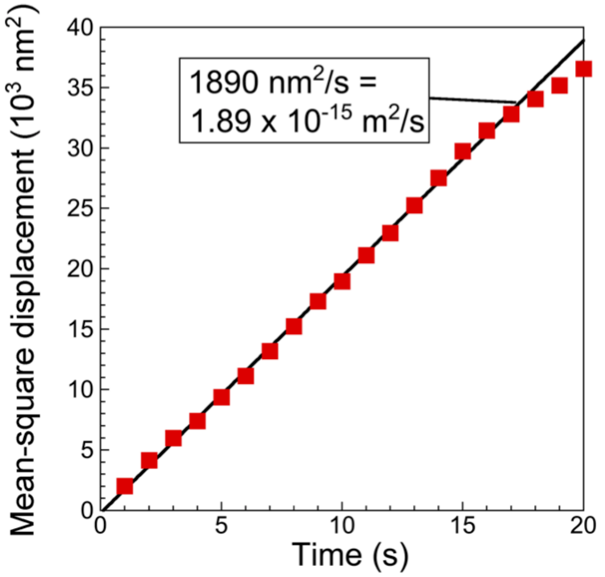

Supplementary Figure 16. Motion of a individual octapods in toluene. Mean-square displacement as a function of time, determined from a series of in-situ STEM images.

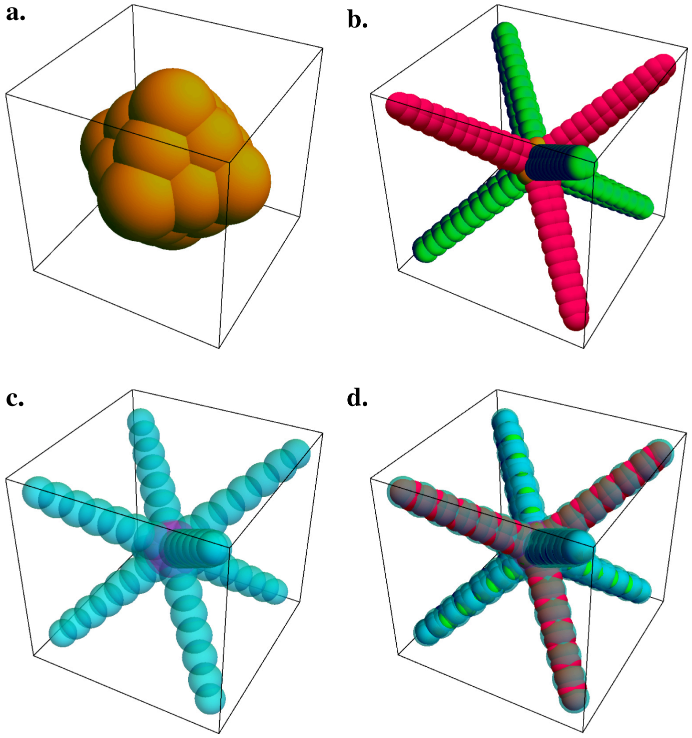

Supplementary Figure 17. Representation of the coarse-grained model for the octapod-shaped branched colloidal nanocrystals considered in the experiment. a. Hard octahedral core of the octapod, consisting of 18 Molecular Dynamics (MD) beads (gold) that interact via a Weeks-Chandler-Anderson (WCA) interaction potential. **b.** The hard part of the octapod: core (gold) and pods (green, red). Each pod consists of 34 MD beads, which also interact via a WCA interaction potential. The beads are arranged in a triangular configuration that tapers towards the tip, to reproduce the triangular shape observed in the experiment. The use of color is to differentiate between pods that (may) have different physical properties, e.g., positive and negative charge. **c.** Interaction backbone of the model. The backbone consists of 57 MD beads, which may be endowed with different generic Lennard-Jones interaction potentials or charges; the central bead is indicated in transparent magenta and the pod beads using transparent cyan. **d.** The full model for octapods, which interact via the calculated van der Waals interactions. The radius of the soft beads is chosen according to the range of the beads' interaction potential.

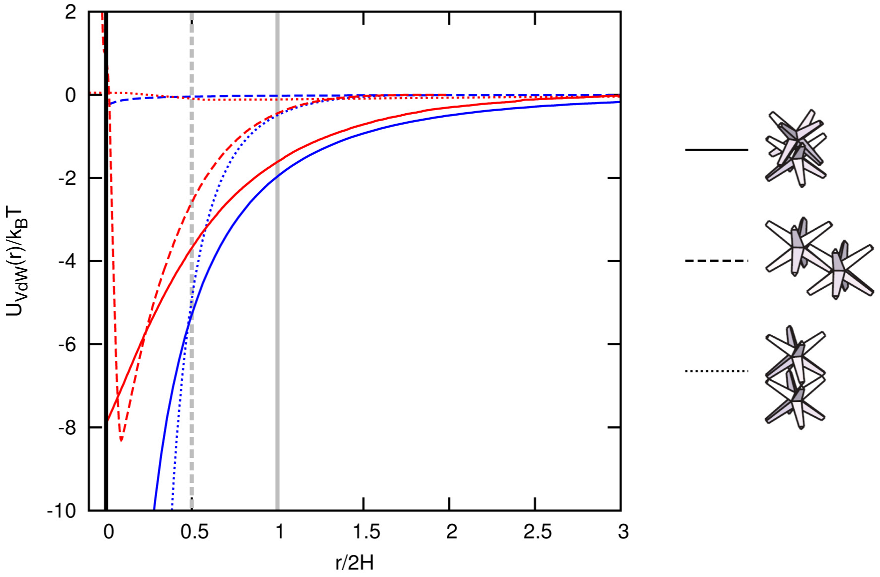

Supplementary Figure 18. The distance-dependence of the total interaction potential between two model octapods for various configurations representative of those found in experiment. The total interaction potential from our simulation model A (red) and from the theoretical calculation of van der Waals (vdW) interaction $U_{\mathrm{vdW}}(r)$ (blue) are shown, also see Ref. [1]. Here, the interaction between the octapods is given as a function of the distance between the surfaces r of two octapods that are initially in contact ($r = 0$) (thick black line) and move away from each other along the line connecting their centers. The contact point of the simulation model A is defined as the value of the octapod separation, for which the interaction potential is +1 $k_B T$, with k_B Boltzmann's constant and T the temperature; set to $r = 0$ here. H is the length of the ligands that form the capping layer (2.5 nm), which covers the octapods and induces steric repulsion, see Ref. [1]. The thick gray lines indicate the distance for which the octapods' surfaces are separated by one and two capping lengths, respectively. We considered three configurations for which we checked the correspondence between the vdW calculation and the simulation result: interlocking (solid curves), pod-pod contact (dashed), and tip-tip contact (dotted). Note that agreement between the simulation model A and the theory is reasonable when $r > 2H$, i.e., outside the range of steric interactions.

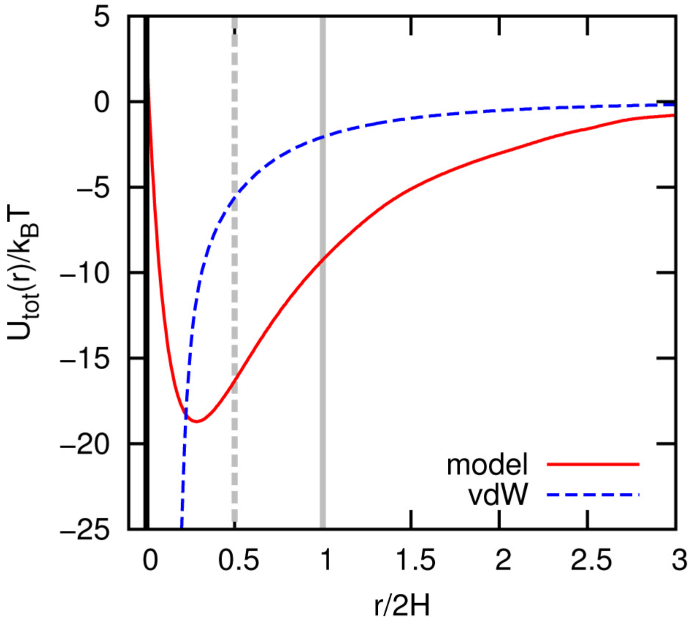

Supplementary Figure 19. The interaction potential required to achieve interlocking-chain self-assembly in our molecular dynamics simulations. The total interaction potential $U_{tot}(r)$ between two octapods from our simulation model B (red, solid) is compared to the theoretically calculated van der Waals (vdW) interaction (blue, dashed). The notation is the same as in supplementary Fig. 18.

Supplementary Note 1. Additional Evidence for a Wet Environment during 1D Chain Self-Assembly

The continued diffusion of monomers in all parts of the FOV throughout the observation period provides evidence that the liquid cell remains filled with toluene solvent at all times (Supplementary Fig. 8; Supplementary Movie 7). The STEM contrast between the high- and low-concentration regions in Fig. 2 of the main text could be interpreted as a liquid-gas interface, however, such an interface was not observed anywhere in the viewing window of the liquid cell, neither before nor after the appearance of the octapod chains. Indeed, a quantitative analysis of low-loss electron energy loss (EELS) spectra obtained at different points of the liquid cell show that all parts of the viewing window are filled with liquid, whose thickness varies between 90 nm and 190 nm (Supplementary Figs. 3-6) and in which diffusion and rotation of suspended octapods is observed even in the thinnest areas (Supplementary Fig. 3). In the areas in which the self-assembly of linear octapod chains is observed the liquid thickness was greater than 140 nm, both before and after the assembly process. The fact that the liquid thickness remained unchanged over the period of observation of assembly in several areas of the cell (> 1 hour) strongly supports the notion that our experiments do not involve any significant drying of the solvent (Supplementary Fig. 5).

Hence, the transition seen in Fig. 2 reflects merely a boundary between areas of high and low octapod density. Analysis of the contrast in STEM further supports this conclusion: initially, there is a clear difference in contrast (as perceived visually) between the high- and low-concentration regions (Supplementary Fig. 9 a, b). As octapod chains form in the high-concentration region, the STEM contrast in their vicinity changes sharply and becomes nearly identical to that in the low-concentration region (Supplementary Fig. 9 c, d), because the density of high-Z objects suspended in the solution drops as octapods self-assemble into chains. The residual area in which individual suspended octapods can still be found thus shrinks progressively toward the lower boundary of the FOV. In absence of any significant drying of the solvent, the overall directionality of the self-assembly may be due to the drop in the local concentration of free octapods accompanying chain formation. As newly formed chains deplete their surrounding solution of octapods, this apparently provides an environment that facilitates self-assembly in their vicinity. This, together with our observation that no chains form in low-concentration areas, suggests the existence of an optimal concentration at which self-assembly proceeds most efficiently.

Supplementary Note 2. Brownian Motion of Individual Octapod Nanocrystals in Toluene

By effectively creating a '2.5D' (i.e., half-space) observation environment, the SiN_x membranes of the liquid cell enable high-resolution imaging of self-assembly without significantly altering the underlying solution-phase processes. We used real-time observations of the Brownian motion of individual octapods near the membrane to further characterize this environment. Following individual octapods over long distances is difficult due to frequent jumps outside the field of view,[2,3] or jumps resulting in attachment to chain segments. To suppress these events, we reduced the liquid thickness in the cell compared to the experiments discussed previously. We expect the measured diffusion coefficient to represent a lower bound to that in the thicker cells used for studying self-assembly. Individual octapods move both by translation and rotation. From the mean-square displacement of the translational motion, which is clearly Brownian (Supplementary fig. 16), we estimate a diffusion coefficient for octapods in toluene, $D_t \approx 1 \cdot 10^{-15}$ m^2 s^{-1}. The expected translational diffusion coefficient for an octapod with an estimated hydrodynamic radius of 40 nm in a bulk fluid is $D_t \approx 7 \cdot 10^{-12}$ m^2 s^{-1}, i.e., 3 orders of magnitude larger than the experimentally determined one. This difference can be explained by a reduced diffusivity due to the interaction of the octapods with the SiN_x membrane. However, in our case these membrane interactions are relatively weak, compared for example to the significantly dampened dynamics and much lower (10^{10} times) diffusion coefficients found previously for the movement of particles in ultrathin liquid layers.[4]

Supplementary Note 3. Statistical Mechanical Model

The relative abundance of n-octapod chains in equilibrium can be determined from the grand canonical distribution function:

$$C_n \propto \int \left(\prod_{i=1}^{n-1} \frac{d\Omega_i d\psi_i d\vec{r}_i}{8\pi^2} \right) e^{-\beta[H-\mu n]} \quad \textbf{(1)}$$

Here the integration goes over translational and rotational degrees of freedom of $n-1$ octapods (which are internal degrees of freedom on the chain, with $8\pi^2$ representing the result of integration over all Euler angles of an unconstrained free octapod. H is the interaction Hamiltonian of the system, $\beta = 1/kT$ and μ is the chemical potential of single (unbound) octapods that depends logarithmically on their density: $\mu = kT \ln C_1$.

It is a formidable task to calculate the above quantity precisely, even if the interaction potential is known. Instead, we will use a number of simplifications, which, however, will preserve the crucial physical aspects of the problem. First, let us consider a case where the interactions are so strong that the system has a maximal possible number of contacts between octapods, Z_n. This implies that out of $6(n-1)$ internal degrees of freedom, Z_n are strongly localized due to an interaction potential acting between the

pods. First, we can conduct integration over those degrees of freedom, which can be simply represented by a set of relative separations of the pairs of pods in contact, x_{ij}. The result of this integration can be expressed as

$$\int dx_{ij}\, e^{-\beta V(x_{ij})} = \delta e^{\beta\epsilon} \qquad \textbf{(2)}$$

Here $-\epsilon$ is the depth of the interaction potential and δ is a characteristic localization length that arises from the integration around that minimum. Since the integration variables, x_{ij}, are different from the original degrees of freedom, one needs to introduce a proper Jacobean. In order to estimate it, we note that replacing one translational degree of freedom with one bond length results in a factor of the order of 1. It can be estimated in the following manner. If one shifts a specific octapod by a vector $d\vec{r}_i$ and rotates it by a small angle, $d\vec{\theta}_i$, the bond length between that octapod and its neighbor changes by the amount

$$dx_{ij} = -\hat{n}_{ij} \cdot \left(d\vec{\theta}_i \times \vec{R} + d\vec{r}_i\right) \qquad \textbf{(3)}$$

where $\vec{R}$ is the position of the i-j contact with respect to the center of the i-th octopod, and $\hat{n}_{ij}$ is a unit vector along the bond between the two pods in contact. Based on this expression, if we replace one translational or one rotational degrees of freedom with the bond length x_{ij}, the overall expression for the abundance of the n-chains gains a factor of the order of 1 or 1/r, respectively. This leads to the following result:

$$C_n \sim C_1^n \frac{\Delta^{6(n-1)-Z_n}}{R^{3(n-1)}} \left(\delta e^{\beta\epsilon}\right)^{Z_n} \qquad \textbf{(4)}$$

Here the factor Δ represents an integration of each translational degree of freedom not suppressed by the binding. Another important aspect of this calculation is that due to the cubic symmetry of an octapod, it has $6\times4 = 24$ equivalent orientations (just like a cube). Since the integration over bond length only accounts for one specific binding topology, the overall distribution has to be multiplied by $24^{(n-1)}$. This correction largely compensates the role of the prefactor $[8\pi^2]^{-(n-1)}$ in the original expression. The remaining factor of the order of 1 per degree of freedom, can be absorbed e.g. within the model parameter R.

Our final step is to account for the fact that the chain formation does not require individual bonds to be very strong. Instead, each of them can break and re-form without the octapod chain losing its integrity. In fact, this increases the chain stability due to an additional entropy. In the approximation that each of the breaking events is independent of the others, this gives rise to a minimal modification of our previous result: we need to replace each factor $\delta e^{\beta\epsilon}$ with $\Delta + \delta e^{\beta\epsilon}$. Here Δ again represents an integration over a translational degree of freedom that emerges after the bond is broken. This leads to the result presented in the main text of the paper:

$$C_n \sim \left(\frac{C_1 \Delta^6}{R^3}\right)^{n-1} \left(1 + \frac{\delta}{\Delta} e^{\epsilon/kT}\right)^{Z_n} \quad \textbf{(5)}$$

Supplementary Note 4. Interlocking Chains using Molecular Dynamics Simulations

In this section, we discuss the results of the MD simulation models A and B (see Supplementary Methods 1 below) and relate these to the experimental results, as well as the theoretical calculations performed in the main manuscript.

Model A, Van der Waals Interactions. We consider the interlocking configuration, to relate model A to the theoretical calculations performed in our manuscript. The dimer (interlocking) configuration has 8 pod-pod contacts, because we used a perfectly symmetric octapod with perfect alignment between two octapods to determine the vdW interaction energy. Therefore, the value of the energy per contact at 2.5 nm separation ($r = H$) is about 0.5 $k_B T$. If we assume that this configuration corresponds to a four contact situation, we obtain about 1 $k_B T$ of energy per contact in the simulation model. This is too low to observe self-assembly according to the theoretical model.
Indeed, using the procedures described in Supplementary Methods 1, we did *not* observe self-assembly in any of the simulations that we performed. We performed 10 simulations for each of the number of octapods in the box, thus allowing us to obtain reasonable statistics for a range of particle densities. It should be noted that the particle density $\phi = N/L^3$ for each of the N that we simulated, is in the range of the preparation concentrations used in the experiment, namely: 10^{-8} mol l^{-1}. A single simulation of 20 octapods at a density comparable to the one in the corner of the cell, where the self-assembly was experimentally observed, did not result in interlocking-chain formation either. The 30% difference between the energy of interlocking configuration calculated in Ref. 5 at $r = H$ and the one used in our model A, see supplementary Fig. 18, is insufficient to effect the conclusion that vdW-type interactions are sufficient to induce self-assemlby of the octapods.

Model B, Short-Ranged Beyond-vdW Interactions. For this interaction, we observed self-assembly into interlocking chains. Our samples contained mostly interlocking chains and only occasionally branched structures, which did not appear long-lived. For greater numbers of particles in the box, mixtures of short and long chains with minimal branching were observed. The maximal chain length for these parameters was found to be 9, longer chains showed a tendency to break. It is therefore likely that these interactions are of sufficient range and strength to account for the self-assembly observed in the experiment. However, we did not observe preferential self-assembly of trimers and limited self-assembly of dimers using this model. In fact, dimers are the first step in the formation of longer oligomers of octapods in these simulations. This indicates that the

essential physics of the system is not captured by our simulation model, presumably due to a mismatch in directionality of the interaction potential compared to the experimental system, as well as difficulties in achieving the time scales of the experiment.

In summary a substantially longer-ranged interaction potential is required to achieve self-assembly in our simulations than the vdW interaction predicted by our Hamaker-de-Boer calculations.[1] In fact, the decay length of the potential had to be increased by a factor of three for the interlocking configuration to occur, see supplementary Fig. 19. The interaction potential also had to be substantially deeper than we estimated from our previous predictions. This is in agreement with the theoretical model put forward in the main text, which predicts a contact-point interaction strength of ~2.7 kT for the experimentally observed self-assembly.

In the experiment a preferential self-assembly of trimers into the interlocking configuration was observed, while stable interlocking dimers were not found. This observation was explained by our theoretical model, and it, together with the above simulation results, allowed us to exclude vdW interactions as the main driving force behind the observed self-assembly. However, excluding a vdW-only based mechanism leaves significant freedom on how to achieve the desired self-assembly within the simulation model. Indeed, a combination of vdW, steric, Coulomb, and other interaction potentials may be at play in our system.

Supplementary Methods. Molecular Dynamics Simulations

In this section, we present the simulation methods used to study the self-assembly observed in the *in-situ* transmission electron microscopy (TEM) study of octapod-shaped branched nanocrystals (bNCs) described in the main manuscript. We implemented two simple coarse-grained models for the octapod bNCs, labelled A and B, as detailed below. These models are based on Molecular Dynamics (MD) simulations with Langevin dynamics, which were performed using the MD software package **E**xtensible **S**imulation **P**ackage for **Res**earch on **So**ft Matter (*ESPResSo*).[5]

Generic Coarse-Grained Octapod Model. Anisotropic hard-core interactions and short-ranged attractive van der Waals (vdW) interactions are considered crucial for the formation of the interlocked structures in bulk suspension, based on our previous study of the octapod self-assembly.[1] Therefore, we coarse-grained the shape and theoretically predicted interactions of the octapods to achieve a model suited for MD simulations. In Refs. [1,6,7] we approximated the shape of the octapod by a triangular mesh and a spherocylindrical model, respectively, in order to endow the model with a hard-core interaction. In this study it proved more convenient to construct our model using a combination of isotropic interaction potentials arranged in an anisotropic way to achieve the same effect, see Supplementary Fig. 17. These interaction potentials are as follows

$$U_{ij}^{WCA}(r) = \begin{cases} \min\left[20k_BT, -40k_BT\left(\left(\frac{\sigma}{r}\right)^{12} - \left(\frac{\sigma}{r}\right)^{6} - 10k_BT\right)\right] & r < 2^{1/6}\sigma \\ 0 & r \geq 2^{1/6}\sigma \end{cases}, \quad \textbf{(6)}$$

$$U_{ij}(r) = \begin{cases} \varepsilon_{ij}\left(\left(\frac{R_{ij}}{r}\right)^{m_{ij}} - \left(\frac{R_{ij}}{r}\right)^{n_{ij}} + s_{ij}\right) & r < c_{ij} \\ 0 & r \geq c_{ij} \end{cases}, \quad \textbf{(7)}$$

In the representation shown in Supplementary Fig. 17 the various parts of our model have been color coded to indicate differences in the employed interaction potentials. Our model consists of 347 so-called MD beads. The hard core, see supplementary Fig. 17 (a, b) is composed of 290 MD beads which interact with beads of the same type via a Weeks-Chandler-Anderson (WCA) potential, which in our case is defined by Eq. (6), where r is the inter-bead distance in units of s, k_B is Boltzmann's constant, and T the temperature. Note that we cap our potential at 20 k_BT in order to allow for the WCA beads to overlap to better match the triangular cross section of the pods, without this leading to extremely high energies. The 57 beads belonging to the ranged-interaction backbone, see Supplementary Fig. 17 c, interact with each other according to the generic Lennard-Jones (LJ) interaction potential specified in Eq. (7), where i, j are the indices of the bead types; e_{ij} gives the strength of the interaction potential in units of k_BT; R_{ij} is a measure for the size of the particle in units of s; m_{ij} and n_{ij} are exponents that give the decay of the repulsive and attractive part of the potential, provided $m_{ij} > n_{ij} > 3$, respectively – the latter constraint implying a short-ranged and truncateable potential; s_{ij} is a shift in the value of minimum of the potential and is dimensionless; and finally c_{ij} is the range at which the potential is cut, which also has units of s. To ensure that Newton's third law is obeyed all coefficients are symmetric under exchange of i and j. The values of the above parameters were tuned to ensure that the theoretically predicted vdW attractions are accurately reproduced, as we will detail shortly. All beads are connected to a central bead via a rigid virtual bond,[5] such that the entire octapod retains its shape during the simulation. The forces and torques are translated back to this central bead. This creates a rigid structure that is free to translate and rotate. MD beads from one octapod do not bond with the central bead of another.

Our change in modeling from triangular-tessellation (Refs. [1,6,7]) to cluster-based should not lead to an oversimplification of the hard-core interaction between octapods, since we have already demonstrated that the use of spherocylindrical elements leads to qualitatively similar behavior for this interaction compared to that of a triangular-mesh-based model.[6,7]

Parameter Mapping. In order to compare simulation models to the vdW calculations of Ref. [1,6,7], we mapped the MD units onto the physical units using experimentally reasonable parameters. The effective hard-core arm length of our model is $L = 5.5\ \sigma$ and the length of the arms in the experimental system is ~60 nm, therefore the MD base unit of length $\sigma \approx 11$ nm. The liquid in which the octapods are suspended is assumed to be at room temperature, i.e., 300 K, from which we obtain the value for the MD base unit of energy of $\varepsilon \equiv k_B T \approx 4.1 \cdot 10^{-21}$ J. The final ingredient required to fix all the MD unit conversions is the mass of a cube with volume σ^3, or, equivalently, the density of the material. We chose the density to be that of CdSe: $\rho = 5.8 \cdot 10^3$ kg m^{-3}, which sets the MD base unit of mass to $m = 7.7 \cdot 10^{-21}$ kg. These three units, length, energy, and mass, allow us to calculate the MD base unit of time: $\tau = \sqrt{m\sigma^2/\varepsilon} \approx 1.5 \cdot 10^{-8}\,\mathrm{s}$. Using this mapping we were able to quantify the difference between our simulation model and the vdW interaction calculated in our previous work.[1]

Matching Molecular Dynamics Parameters to Experiment. We tuned the parameters for the simulations to model the experimental system. We started by introducing a mass and inertia tensor, in order to accurately capture the short-time dynamics of the particles. The mass was computed by integrating the density over the volume covered by the WCA beads (accounting for the overlaps); resulting in a total mass of $M = 38\ m$. We used standard integral expressions to determine the moment of inertia over the same volume. This resulted in a diagonal inertia tensor with equal entries on the diagonal of $I = 2.0 \cdot 10^2\ m\,\sigma^2$.

We also calculated the hydrodynamic radius of the octapod in order to estimate the diffusion constant of these particles. The expression for the hydrodynamic radius of an assembly of spheres[8] was used to approximate the value of the octapod's hydrodynamic radius and we found $R_H \approx 3.7\ \sigma$. The translational diffusion constant that follows from R_H is $D_T = k_B T/(6\pi\eta\ R_H) = 1.2 \cdot 10^{-3}\ \sigma^2\tau^{-1}$, where we used the value of the viscosity of toluene $\eta = 5.5 \cdot 10^{-4}$ kg m^{-1}s^{-1} or equivalently $\eta = 12\ m\sigma^{-1}\tau^{-1}$ in MD units. N.B. This is the bulk value of the translational diffusion coefficient; the diffusion coefficient under confinement may be substantially reduced, as in the experiment the translational diffusion coefficient was determined to be 1000 times lower.

From the above, we can determine the friction that is required in the Langevin thermostat to ensure that the long-time diffusion matches the estimated experimental value. A back-of-the-envelope calculation indicated that only thermostating the central bead, as is typical for virtualized (rigid) models, would lead to a prohibitively large friction coefficient $\zeta \approx 8 \cdot 10^2\ m\tau^{-1}$. Such a high value negatively impacts the stability of the MD simulation, thus necessitating an extremely small time step. Moreover, it effectively stretches the range of the ballistic regime of the particle diffusion, giving rise to

erroneous short-time behavior. We employed the features `VIRTUAL_SITES_THERMOSTAT` and `THERMOSTAT_IGNORE_NON_VIRTUAL` of *ESPResSo*[5] to thermostat the 346 virtual $n > 0$ beads instead of the single central bead to reduce the friction coefficient.

The friction coefficient was tuned to achieve the desired diffusion coefficient by performing short simulations, wherein we calculated the mean square displacement (MSD) and velocity auto-correlation function (VACF) of a single octapod in a cubic simulation box of length (100 σ^3) with periodic boundary conditions. A Langevin thermostat 'temperature' of 1.0ε and a friction coefficient of $\zeta = 1.75\ m\tau^{-1}$ resulted in a long-time diffusion of $D_T = 1.2 \cdot 10^{-3}\ \sigma^2\tau^{-1}$. We were restricted to using an MD time step of 0.01τ to ensure stability of the MD integration. This implies that $6.7 \cdot 10^6$ integration steps go into simulating 1 second of 'real' time. N.B. Henceforth, we will express the number of integration steps used in the simulation in terms of the effective real time, e.g., 6705 steps correspond to 1 ms. This also allows for direct comparison to the acquisition time given in the main manuscript when we discuss our results.

Simulation Approach. To verify if our vdW-based theoretical model could produce chains, we considered self-assembly in three-dimensional (3D) systems, rather than in the more complex quasi-two-dimensional (quasi-2D) environment of the octapods confined in the *in-situ* TEM fluid flow cell. In particular, we focused on a small number of octapods $N = 2$ to 6 in a cubic simulation box with periodic boundary conditions and edge length of $L = 40\sigma$. These octapods were initialized at random positions throughout the box and were allowed to reposition and orient randomly to prevent overlaps via an initial warm-up integration (100 ms). During this warm up, the generic LJ potentials were not activated. We then switched on the generic LJ potentials and equilibrated the system for an additional 100 ms. The typical production run length was 1 s, which is substantially shorter than the experimental time scale, but was close to the limit of what is acceptable in terms of CPU hours.

Model A. We matched the coefficients (ε_{ij}, R_{ij}, m_{ij}, n_{ij}, s_{ij}, and c_{ij}) to most accurately reproduce the vdW interactions obtained in Ref. 1. Supplementary Fig. 18 shows the result as a function of the distance for three configurations, where we show both our simulation model and the result of our vdW calculations. In Ref. [1] we argued that the repulsive interaction between octapods at close separation is governed by a steric contribution. However, we were uncertain about the local surface coverage of polymers that effect this repulsion. We therefore estimated the depth of the minimum from the value of the vdW interaction at surface separations comparable to the length of the fully stretched polymer, $H = 2.5$ nanometers (see Ref. 1); where we assumed that for larger distances the steric repulsion would be dominated by vdW attraction. We arrived at a potential well depth of around 3 to 4 k_BT in toluene for the interlocking configuration.

From Supplementary Fig. 18 it becomes clear that outside of the range where steric interactions can play a role ($r > 2H$), the agreement between our theoretical vdW result and the simulation model is quite excellent. Our model has a slightly deeper potential well for the interlocking configuration of around 8 $k_B T$, though.

Model B. We also used a model with different coefficients (ε_{ij}, R_{ij}, m_{ij}, n_{ij}, s_{ij}, and c_{ij}) to increase the strength and range of the interactions between the beads belonging to the backbone. This allows us to account for interactions that go beyond the vdW forces calculated in Ref. 1. Supplementary Fig. 19 shows a comparison between the calculated vdW interaction for the interlocking configuration and the one used to achieve self-assembly with our 'improved' model B. To relate this model to the theoretical calculations, we again consider the configuration for which we computed the difference in Supplementary Fig. 19, as a perfectly interlocking one with 8 pod-pod contacts. This gives us a value of approximately 2 $k_B T$ per contact, which is much closer to the 2.7 $k_B T$ predicted by the theory, especially when we factor into this that the interaction is longer ranged as well.

Supplementary References

1. Miszta, K., de Graaf, J., Bertoni, G., Dorfs, D., Brescia, R., Marras, S., Ceseracciu, L., Cingolani, R., van Roij, R., Dijkstra, M. & Manna, L. Hierarchical self-assembly of suspended branched colloidal nanocrystals into superlattice structures. *Nature Mater.* **10**, 872-876 (2011).
2. Jungjohann, K. L., Bliznakov, S., Sutter, P. W., Stach, E. A. & Sutter, E. A. In Situ Liquid Cell Electron Microscopy of the Solution Growth of Au–Pd Core–Shell Nanostructures. *Nano Lett.* **13**, 2964-2970 (2013).
3. Sutter, E., Jungjohann, K., Bliznakov, S., Courty, A., Maisonhaute, E., Tenney, S. & Sutter, P. In-situ Liquid Cell Electron Microscopy of Ag-Pd Galvanic Replacement Reactions on Ag Nanoparticles. *Nat. Commun.* **5**, 4946-4954 (2014).
4. Lu, J. Y., Aabdin, Z., Loh, N. D., Bhattacharya, D. & Mirsaidov, U. Nanoparticle Dynamics in a Nanodroplet. *Nano Lett.* **14**, 2111-2115 (2014).
5. Limbach, H. J., Arnold, A., Mann, B. A. & Holm, C. ESPResSo—an extensible simulation package for research on soft matter systems. *Comput. Phys. Commun.* **174**, 704-727 (2006).
6. Qi, W., de Graaf, J., Qiao, F., Marras, S., Manna, L. & Dijkstra, M. Phase diagram of octapod-shaped nanocrystals in a quasi-two-dimensional planar geometry. *J. Chem. Phys.* **138**, 154504-154501 - 154504-154513 (2013).
7. Qi, W., Graaf, J. d., Qiao, F., Marras, S., Manna, L. & Dijkstra, M. Ordered Two-Dimensional Superstructures of Colloidal Octapod-Shaped Nanocrystals on Flat Substrates. *Nano Lett.* **12**, 5299-5303 (2012).
8. des Cloizeaux, J. & Jannink, G. *Polymers in solution: Their modeling and structure.* Trans. rep. edn, (Oxford University Press, 1991).
